# Synthesis and reactivity of aliphatic sulfur pentafluorides from substituted (pentafluorosulfanyl)benzenes

**DOI:** 10.3762/bjoc.12.12

**Published:** 2016-01-20

**Authors:** Norbert Vida, Jiří Václavík, Petr Beier

**Affiliations:** 1 Institute of Organic Chemistry and Biochemistry, v.v.i., Academy of Sciences of the Czech Republic, Flemingovo nám. 2, 166 10 Prague 6, Czech Republic; 2 Laboratory of Molecular Structure Characterization, Institute of Microbiology, v.v.i., Academy of Sciences of the Czech Republic, Vídeňská 1083, 142 20 Prague, Czech Republic

**Keywords:** dearomatization, decarboxylation, Diels–Alder reaction, oxidation, pentafluorosulfanyl group

## Abstract

Oxidation of 3- and 4-pentafluorosulfanyl-substituted anisoles and phenols with hydrogen peroxide and sulfuric acid provided a mixture of SF_5_-substituted muconolactone, maleic, and succinic acids. A plausible mechanism for the formation of the aliphatic SF_5_ compounds was presented and their chemical reactivity was investigated. SF_5_-substituted *para*-benzoquinone was synthesized; its oxidation led to an improved yield of 2-(pentafluorosulfanyl)maleic acid. The reaction of SF_5_-substituted maleic anhydride and *para*-benzoquinone with cyclopentadiene afforded the Diels–Alder adducts. Decomposition of 3-(pentafluorosulfanyl)muconolactone in acidic, neutral and basic aqueous media was investigated and the decarboxylation of 2-(pentafluorosulfanyl)maleic acid provided 3-(pentafluorosulfanyl)acrylic acid.

## Introduction

**Scheme 1:**
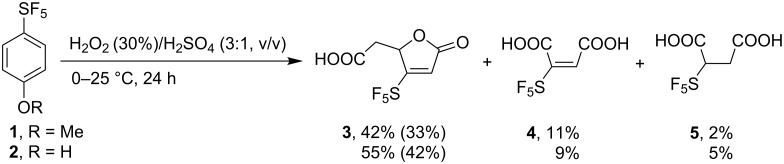
Oxidation of SF_5_-anisole and phenol. ^19^F NMR yields are shown (isolated yields in parentheses).

Fluorinated organic compounds have been one of the foci of chemical industry for the last several decades. The unique properties of fluorine atoms and fluorinated groups have been exploited in various applications ranging from advanced materials to bioactive compounds [1–3]. One such fluorinated functional group which has become the focus of systematic investigation only recently is the pentafluorosulfanyl (SF_5_) group [4–5]. The combination of high stability [6], lipophilicity [7] and strong electron-withdrawing character [8] – similar to but more extreme than the trifluoromethyl group – makes SF_5_ compounds promising candidates for agrochemicals and pharmaceuticals [9]. Recent studies showed several cases where SF_5_ analogues of established compounds showed improved potency [10–12]. Limiting factors for a more widespread use of SF_5_ compounds are low accessibility of basic building blocks and the lack of understanding of their chemical behavior. Lack of availability of SF_5_ compounds is particularly evident in aliphatics where radical addition of expensive and toxic SF_5_Cl to unsaturated compounds is practically the only synthetic option currently available [13–14]. We have recently described an alternative route to access SF_5_ aliphatic compounds, based on oxidation of much more readily available aromatic ones [15]. One of the methods we developed was based on the oxidation of 4-(pentafluorosulfanyl)anisole (**1**) or 4-(pentafluorosulfanyl)phenol (**2**) by a mixture of aqueous hydrogen peroxide and concentrated sulfuric acid (Scheme 1). The major product was muconolactone **3** while maleic acid **4** and succinic acid **5** were formed in small amounts. Herein we report a full account of this oxidation, alternative syntheses of maleic acid **4**, and the results of chemical reactivity studies of acids **3** and **4**.

## Results and Discussion

**Scheme 2:**
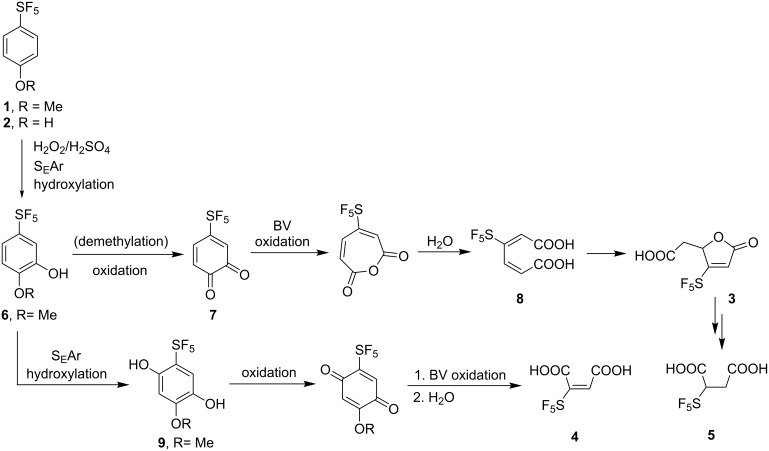
Proposed mechanism for the formation of **3** and **4** from SF_5_ aromatics **1** and **2**.

The proposed mechanism for the oxidation of **1** and **2** to SF_5_-containg products is shown in Scheme 2 [15]. The muconolactone derivative **3** is most probably the product of a series of transformations: electrophilic aromatic hydroxylation to the intermediate **6**, oxidation to *ortho*-benzoquinone **7**, Baeyer–Villiger (BV) oxidation, hydrolysis to muconic acid derivative **8**, and finally intramolecular conjugate addition affording **3**. Under certain conditions, small amounts of intermediates **6** and **8** were detected [15]. Interestingly, this reaction sequence is essentially identical to the aerobic microbial degradation of aromatic compounds, which involves hydroxylating oxygenase and ring-cleaving dioxygenase enzymes [16–19]. This sequence of reactions however, does not account for the presence of maleic acid derivative **4** in the product mixture. Control experiments in which **8** or **3** were reacted with H_2_O_2_/H_2_SO_4_ did not provide **4**. This suggests that **4** may be formed by a second hydroxylation of **6** to **9**. The second electrophilic aromatic hydroxylation is a favorable process because **6** is a more activated substrate than **1** or **2**. Oxidation, BV oxidation and hydrolysis would afford the diacid **4** (Scheme 2). Succinic acid derivative **5** is another minor product and a control experiment showed that it was formed from **3**. Mechanistic considerations of this process will be discussed later.

**Scheme 3:**
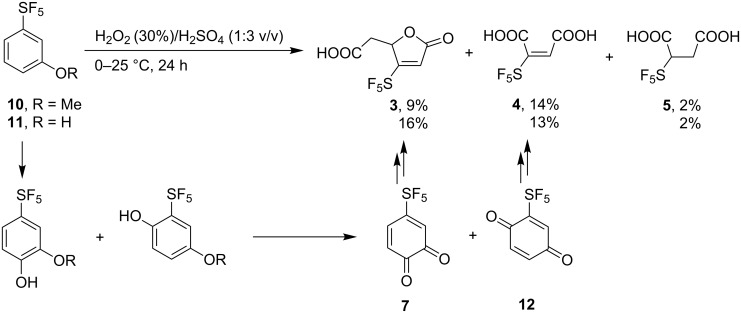
Oxidation of anisole **10** and phenol **11**. ^19^F NMR yields are given.

We hypothesized that the yield of **4** might be increased when starting from 3-(pentafluorosulfanyl)anisole (**10**) or 3-(pentafluorosulfanyl)phenol (**11**). In these substrates, the first hydroxylation is likely to occur in the *para* position to the methoxy or hydroxy groups facilitating the formation of the *para*-benzoquinone derivative **12**. To test this hypothesis, both **10** and **11** were subjected to oxidation by H_2_O_2_/H_2_SO_4_ mixture. Indeed, the yield of **3** decreased considerably while the yield of **4** increased somewhat but still remains low (Scheme 3).

**Scheme 4:**
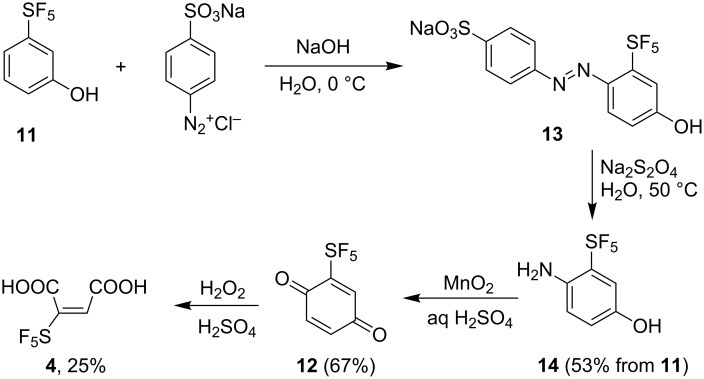
Synthesis of *para*-benzoquinone **12** and oxidation to maleic acid **4**. ^19^F NMR yields are shown, in parentheses isolated yields.

The presumed intermediate 2-(pentafluorosulfanyl)-1,4-benzoquinone (**12**) in the pathway toward **4** was prepared independently (Scheme 4). Azo coupling [20] of phenol **11** with a diazonium salt prepared from sulfanilic acid afforded red azo product **13**. The regioselectivity is governed by the strong *para*-directing hydroxy group despite the presence of the bulky SF_5_ group. Compounds with substituents *ortho* to the SF_5_ group are relatively uncommon [21]. Reduction with sodium dithionite [20] provided aminophenol **14** in good overall yield and oxidation with MnO_2_ [22] gave benzoquinone **12** also in a good yield. Further oxidation with H_2_O_2_/H_2_SO_4_ resulted in a mixture of four SF_5_-containing products. Maleic acid **4** was identified in the mixture (25% ^19^F NMR yield, Scheme 4) but chromatographic separation, crystallization or derivatization (CH_2_N_2_) attempts were not successful. Nevertheless, these results show that SF_5_-substituted *para*-benzoquinones are most likely intermediates in the formation of **4** by oxidation of aromatics.

**Scheme 5:**
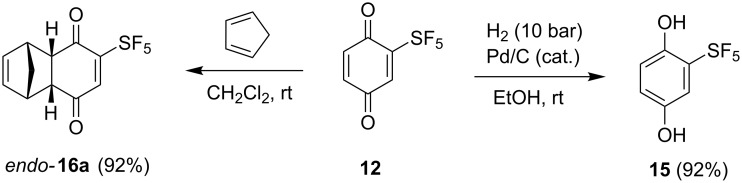
Catalytic hydrogenation and Diels–Alder reaction of benzoquinone **12**.

Benzoquinone **12** is a solid material with good stability at −20 °C. Several standard reactions were investigated. Reduction to hydroquinone **15** using catalytic palladium was very efficient (Scheme 5). The Diels–Alder reaction with cyclopentadiene proceeded in a high yield at ambient temperature with exclusive regiochemistry on the less substituted double bond of **12** and complete *endo* stereochemistry (Scheme 5).

Employing density functional theory (DFT) with the B3LYP functional [23] and the aug-cc-pVDZ basis set [24–25], we calculated the reaction coordinates leading to four possible Diels–Alder products (Table 1). Single-point energies of the transition states suggested that pathway **a** was preferred over **b** (difference of ca. 10–20 kJ/mol). This was further supported by comparison of Gibbs free energy values. Boltzmann distribution was used to calculate theoretical *endo*/*exo* selectivity of **16a** (98:2) from the differences of Gibbs energy of the transition states.

**Table 1:** Calculated reaction coordinates of Diels–Alder reaction of **12** with cyclopentadiene.^a^

energy (kJ/mol)	pathway	*E*_TS_^b^	*E*_P_^c^

single-point (Gibbs) 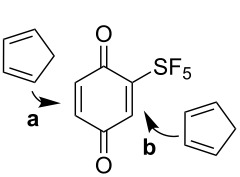	**a***-endo*	53.4 (116.9)	−57.4 (15.9)
	**a***-exo*	64.1 (126.7)	−55.7 (20.1)
	**b***-endo*	74.6 (139.1)	−61.2 (14.8)
	**b**-*exo*	75.6 (140.0)	−58.1 (20.2)

^a^Computed in Gaussian 09 Rev D.01 [26], rB3LYP/aug-cc-pVDZ, gas phase, 298.15 K; ^b^energies of transition states relative to substrates; ^c^energies of products relative to substrates.

**Figure 1:**
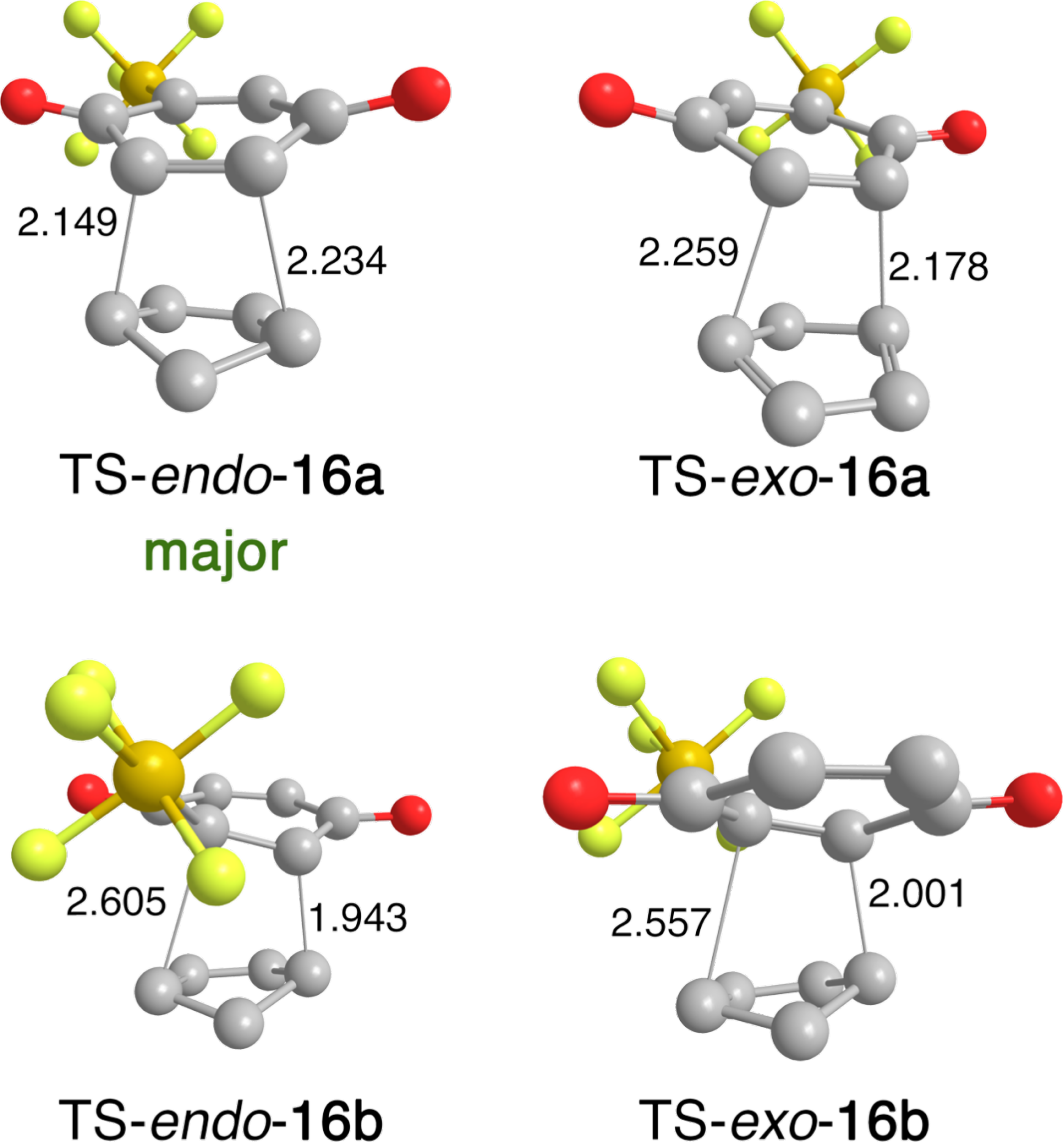
Optimized geometries of transition states of Diels–Alder reaction of cyclopentadiene with **12**. Selected bond lengths are given in Ångström.

Geometries of the four transition states are depicted in Figure 1. The less abundant transition states (TS-*endo*-**16b** and TS-*exo*-**16b**) were asynchronous with the differences of bond lengths of 0.662 and 0.556 Å, respectively. Structures TS-*endo*-**16a** and TS-*exo*-**16a** also displayed a certain degree of asynchronicity with the bond length differences of 0.085 and 0.081 Å. The striking difference is probably caused by the steric demand of the SF_5_ group.

**Scheme 6:**
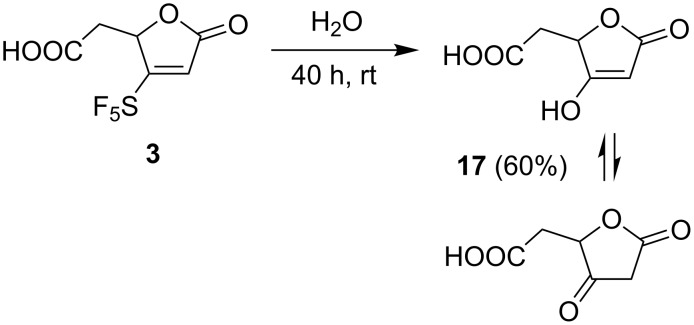
Decomposition of **3** in water.

The chemical behavior of muconolactone **3** was investigated next. This compound showed good stability at ambient temperature in a strongly acidic and oxidizing environment. However, lactone **3** was found to be unstable in water or in aqueous base. Monitoring a solution of **3** in D_2_O by ^1^H and ^19^F NMR spectroscopy showed a gradual disappearance of the SF_5_ signal. After 55 h at ambient temperature two sets of signals appeared in the ^1^H NMR spectrum; their integrals and coupling patterns suggested the presence of two structures of **17** in a 59:41 ratio. In DMSO-*d*_6_ only the enol form of **17** was observed. Decomposition of **3** is much faster in the presence of sodium bicarbonate. In a preparative experiment, **3** was dissolved in water and after 40 h at ambient temperature acid **17** was isolated in 60% yield confirming that **3** undergoes addition of water and elimination of HF and SF_4_ (Scheme 6). Nucleophile-induced elimination of SF_5_ groups in β-position of an electron-withdrawing group has been described [27].

**Scheme 7:**
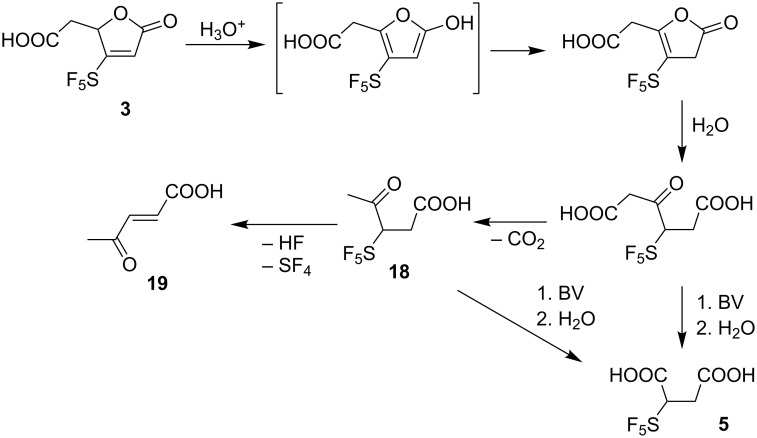
Formation of acids **5**, **18** and **19** from lactone **3**.

As already mentioned, compound **3** is much more stable in acidic than in neutral or basic aqueous medium. To investigate its reactivity in acids under various conditions, several screening experiments were carried out. In HCl (5 M), H_2_SO_4_ (5 M or 10 M) or 85% H_3_PO_4_ at ambient temperature the lactone **3** remained unaffected. At 80 °C the formation of levulinic acid derivative **18** was observed together with unidentified byproducts. Under elevated temperature (100 °C and above) the product of HF and SF_4_ elimination, (*E*)-4-oxopent-2-enoic acid (**19**) [28] formed (Scheme 7). In a preparative experiment using 85% H_3_PO_4_ at 100 °C, levulinic acid **18** was obtained in 14% yield. This result allowed us to propose that byproduct **5** observed during oxidation of SF_5_-anisoles and phenols could be formed from open-chain acids according to Scheme 7.

**Scheme 8:**
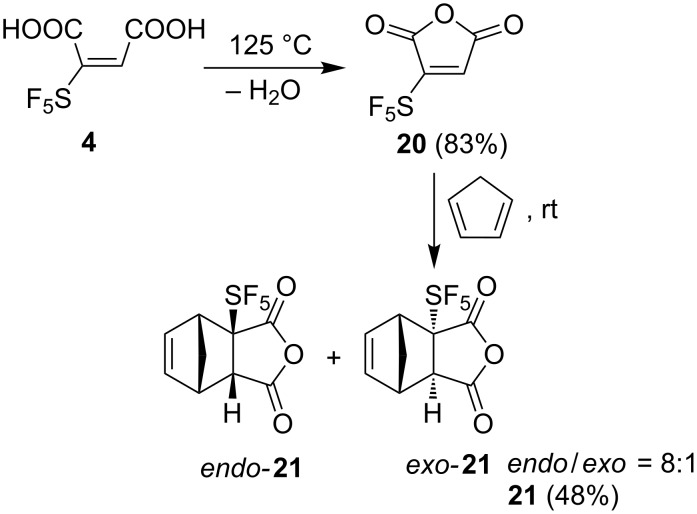
Synthesis of maleic anhydride **20** and Diels–Alder adducts **21**.

Despite the lack of efficient synthetic protocols toward maleic acid **4**, some basic chemical reactivity studies were performed. When heated to about 125 °C under reduced pressure, acid **4** eliminated water to afford anhydride **20** in a good yield. Anhydride **20** underwent a Diels–Alder reaction with cyclopentadiene at ambient temperature to provide a mixture of *endo* and *exo* products in an 8:1 ratio (Scheme 8). In comparison, it is known that maleic anhydride in analogous reactions affords exclusively the *endo* adduct [29].

**Scheme 9:**
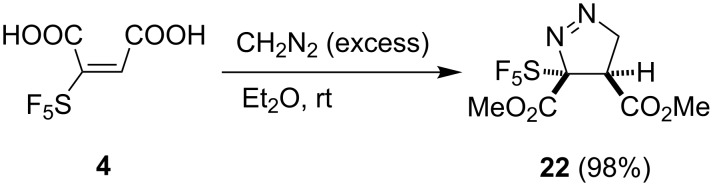
Reaction of maleic acid **4** with diazomethane.

Attempts to facilitate the isolation of very polar maleic acid **4** from the reaction mixture by derivatization to dimethyl ester using diazomethane were not successful. The dimethyl maleate derivative was formed but was accompanied by the [3 + 2] cycloaddition product **22**. With excess of diazomethane, clean and efficient formation of **22** was observed (Scheme 9).

**Scheme 10:**
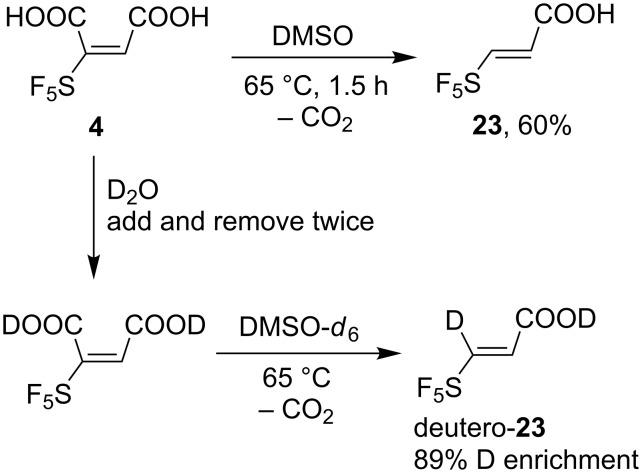
Decarboxylation of maleic acid **4** to acrylic acid **23** in DMSO and the preparation of deuterium labelled **23**.

Acid **4** was stable in water, methanol, isopropylalcohol, boiling benzene, or boiling aqueous sodium carbonate. However, it underwent decarboxylation in DMSO-*d*_6_. Monitoring by NMR showed decarboxylation even at room temperature. After 90 h at ambient temperature the starting compound **4** disappeared and 3-(pentafluorosulfanyl)prop-2-enoic acid (**23**) was formed. At 65 °C the starting material was no longer present after 1.5 h and the NMR yield of the acrylic acid derivative **23** was 60% (Scheme 10). Acrylic acid **23** was prepared previously by the addition of SF_5_Cl to allyl alcohol and subsequent elimination and oxidation [30]. We proposed that the decarboxylation reaction could be used for the synthesis of selectively deuterium labelled acrylic acid derivative **23**. Indeed, when maleic acid **4** was evaporated twice with D_2_O to exchange acidic protons and heated in DMSO-*d*_6_, deuterated **23** was observed by NMR spectroscopy (Scheme 10).

## Conclusion

In conclusion, similar to 4-(pentafluorosulfanyl)anisole and 4-(pentafluorosulfanyl)phenol the *meta*-derivatives **10** and **11** underwent oxidation with aqueous hydrogen peroxide in sulfuric acid to provide SF_5_-muconolactone and SF_5_-maleic acid as main products. Improved conversion of SF_5_-maleic acid was rationalized by preferential formation of SF_5_-substituted *para*-benzoquinone as an intermediate. This benzoquinone was independently prepared and its transformation to the maleic acid derivative by oxidation was verified. Reactions of SF_5_-maleinanhydride (formed by dehydration of SF_5_-maleic acid) and SF_5_-benzoquinone with cyclopentadiene provided the Diels–Alder adducts in good to excellent yields; the observed stereoselectivity of the latter reaction agreed with DFT calculations. SF_5_-muconolactone was found to be relatively stable in acidic media but eliminates the SF_5_ group in water or in aqueous base to form 2-(3,5-dioxotetrahydrofuran-2-yl)acetic acid, which in aqueous solution exists in equilibrium with its enol form. Further experiments are underway to find out whether aerobic microbial degradation of SF_5_-substituted aromatic compounds follows the same pathway as our chemical oxidation method.

## Supporting Information

Synthesis and characterization of all new products, copies of ^1^H, ^13^C, and ^19^F NMR spectra of newly synthesized products, and computational data.

File 1 Experimental part.
