# Supplementary material for: Synthesis and reactivity of aliphatic sulfur pentafluorides from substituted (pentafluorosulfanyl)benzenes
Source: Beilstein J Org Chem. 2016 Jan 20;12:110–6. doi: 10.3762/bjoc.12.12 (PMC4734437; doi:10.3762/bjoc.12.12)
Supplement: File 1 — Experimental part. [file Beilstein_J_Org_Chem-12-110-s001.pdf]

Supporting Information

for

Synthesis and reactivity of aliphatic sulfur pentafluorides from substituted (pentafluorosulfanyl)benzenes

Norbert Vida[1], Jiří Václavík[1,2] and Petr Beier*[1]

Addresses: [1]Institute of Organic Chemistry and Biochemistry, v.v.i., Academy of Sciences of the Czech Republic, Flemingovo nám. 2, 166 10 Prague 6, Czech Republic and [2]Laboratory of Molecular Structure Characterization, Institute of Microbiology, v.v.i., Academy of Sciences of the Czech Republic, Vídeňská 1083, 142 20 Prague, Czech Republic

Email: Petr Beier* - beier@uochb.cas.cz

* Corresponding author

Experimental part

Table of Contents

1. General S2
2. Experimental S2
3. Copies of NMR spectra S7
4. Computational data S31
5. References S43

1. General

IR spectra were recorded on an FTIR instrument using a film technique. NMR spectra were recorded on 400, 500 or 600 MHz instruments at 300 K. The chemical shifts (δ) are reported in parts per million (ppm) and coupling constants (*J*) are given in Hertz. ^{13}C NMR spectra were proton decoupled. The chemical shifts are reported in ppm relative to Me_4Si (0 ppm for ^1H NMR in $CDCl_3$), residual $CHCl_3$ (7.26 ppm for ^1H NMR), residual acetone-d_5 (2.05 ppm for ^1H NMR), residual DMSO-d_5 (2.50 ppm for ^1H NMR), $CDCl_3$ (77.16 ppm for ^{13}C NMR), acetone-d_6 (29.84 ppm for ^{13}C NMR), DMSO-d_6 (39.52 ppm for ^{13}C NMR), and internal $CFCl_3$ (0 ppm for ^{19}F NMR). In several cases, attached proton test (APT) ^{13}C NMR experiments were used to assign carbon signals. Low-resolution MS spectra were recorded using a quadrupole mass-selective electron impact (EI) detector. High-resolution mass spectra (HRMS) were recorded using electron electrospray (ESI) or chemical (CI) ionizations.

2. Experimental

Sodium 4-{[4-hydroxy-2-(pentafluorosulfanyl)phenyl]diazenyl}benzenesulfonate (13). Sulfanilic acid (4.71 g, 27.2 mmol) was suspended in water (28 mL), then Na_2CO_3 (1.44 g, 13.6 mmol) was added in portions. The solution was cooled with ice and an ice cold solution of $NaNO_2$ (2.06 g, 29.9 mmol) in water (4 mL) was added. After about a minute, precipitation started and the suspension was added in portions to a stirred mixture of HCl(aq) (6.2 mL, 35%, 71 mmol) and ice (34 g). After stirring for 45 min in an ice bath, the suspension was added to an ice cold solution of **11** (6.00 g, 27.2 mmol) dissolved in NaOH (aq) (2.19 g NaOH, 55 mmol and 20 mL water). Within seconds, red color formed and red solid precipitated out. The mixture was allowed to stand for 45 min, then NaCl (25 g) was added and the mixture heated to 80 °C. Most of the solids dissolved and the solution was allowed to cool down to RT slowly. The solid was filtered and dried under vacuum to give 15.2 g red solid. ^1H NMR (400 MHz, DMSO-d_6): δ 7.14 (dd, 1H, *J* = 8.9, 2.5 Hz), 7.40 (d, 1H, *J* = 2.5 Hz), 7.74 (d, 1H, *J* = 8.9 Hz), 7.82 (s, 4H); ^{19}F NMR (376 MHz, DMSO-d_6): δ 73.6 (d, 4F, *J* = 153.3 Hz), 87.8-89.6 (m, 1F); HRMS (ESI^-) *m/z* calcd for $C_{12}H_8O_4N_2F_5S_2$ $[M - Na]^-$ 402.9851, found 402.9848.

4-Amino-3-(pentafluorosulfanyl)phenol (14). The crude azo compound **13** (14.3 g) was dissolved in water (80 mL) at 50 °C. To the solution solid $Na_2S_2O_4$ (23.3 g, 134 mmol) was added in portions over 10 min and the temperature was maintained at 50 °C for another 10 min. The color changed from dark red to yellow and solid separated out. The mixture was allowed to cool to RT. The solid was filtered, washed with cold water and dried. Recrystallization of the crude product from boiling benzene (35 mL) gave 3.13 g of **14** as a white solid (53% yield over 2 steps based on **11**); mp 143–144 °C; FTIR (film, acetone) v_{max} (cm^{-1}) 3431, 3413, 3300, 3215, 1617, 859, 832, 810, 802, 797; ^1H NMR (400 MHz, DMSO-d_6): δ 5.22 (s, 2H), 6.76-6.84 (m, 2H), 6.90-6.95 (m, 1H), 9.05 (s, 1H); ^{13}C NMR (100 MHz, DMSO-d_6): δ 112.3 (quin, *J* =

5.0 Hz), 120.6, 121.7, 135.9, 137.0-137.6 (m), 146.5; ^{19}F NMR (376 MHz, DMSO-d_6): *δ* 66.3 (d, 4F, *J* = 148.9), 92.2 (quin, *J* = 149.0 Hz); MS (EI) *m*/*z* (rel. int.) 52 (55), 68 (14), 80 (32), 107 (83), 126 (7), 127 (6), 235 (100); HRMS (CI) *m*/*z* calcd for $C_6H_6F_5NOS$ $[M]^+$ 235.0090, found 235.0091.

2-(Pentafluorosulfanyl)-1,4-benzoquinone (12). Active MnO_2 (1.25 g, 14.4 mmol) was suspended in H_2SO_4 (aq) (15 mL, 2.5 M) at 10 °C. Then 4-amino-3-(pentafluorosulfanyl)phenol (**14**) (1.50 g, 6.38 mmol) suspended in H_2SO_4 (aq) (20 mL, 2.5 M) was added at 10 °C and the mixture was stirred for 75 min at this temperature. The suspension was filtered using a glass filter, the cake was washed with dichloromethane and the aqueous phase extracted with dichloromethane (3 × 20 mL). The organic extract was washed with water (10 mL), NaCl (aq) (6 mL), dried with $MgSO_4$ and evaporated. Chromatography on silica gel, elution with petroleum ether/dichloromethane (1:1, v/v) gave **12** as a yellow solid (1.00 g, 67 %); mp 56-58 °C; R_f 0.44 (petroleum ether/DCM, 1:1); FTIR (film, $CHCl_3$) ν_{max} (cm^{-1}) 1679, 881, 874, 861, 608, 570; ^{1}H NMR (400 MHz, $CDCl_3$): δ 6.86-6.90 (m, 2H), 7.26-7.28 (m, 1H); ^{13}C NMR (100 MHz, $CDCl_3$): δ 135.0, 137.0 (quin, *J* = 4.8 Hz), 137.8 (quin, *J* = 2.7 Hz), 154.8-155.5 (m), 177.3, 185.4; ^{19}F NMR (376 MHz, $CDCl_3$): δ 66.1 (dm, 4F, *J* = 152.0 Hz), 75.8-77.5 (m, 1F); MS (EI) *m*/*z* (rel. int.) 53 (100), 79 (58), 89 (23), 107 (65), 127 (8), 234 (60); HRMS (CI) *m*/*z* calcd for $C_6H_4F_5O_2S$ $[M + H]^+$ 234.9852, found 234.9848.

(Pentafluorosulfanyl)hydroquinone (15). Quinone **12** (40 mg, 0.171 mmol) was dissolved in ethanol (3 mL), 10% Pd/C catalyst (8 mg) was added and the hydrogenation was allowed to proceed at 10 atm H_2, RT. After 3.75 h the catalyst was filtered off, the solvent was evaporated and the residue purified by column chromatography on silica gel in hexane/Et_2O (1:1, v/v). The product **15** was obtained as a white solid (37 mg, 92 %); R_f 0.24 (hexane/Et_2O, 1:1); mp 96-97 °C; FTIR (film, acetone) ν_{max} (cm^{-1}) 3364, 1504, 1444, 844, 814, 601, 577; ^{1}H NMR (400 MHz, acetone-d_6): δ 6.96 (dd, 1H, *J* = 8.9, 2.6 Hz), 6.99 (d, 1H, *J* = 9.0 Hz), 7.18 (dd, 1H, *J* = 2.6, 0.6 Hz), 8.62 (br. s, 2H); ^{13}C NMR (100 MHz, acetone-d_6): δ 115.0 (quin, *J* = 5.3 Hz), 120.4, 121.8, 140.1-140.8 (m), 145.9, 150.1; ^{19}F NMR (376 MHz, acetone-d_6): δ 67.8 (d, 4F, *J* = 148.9 Hz), 86.2-87.9 (m, 1F); HRMS (EI) *m*/*z* calcd for $C_6H_5F_5O_2S$ $[M]^+$ 235.9930, found 235.9931.

***endo*-6-(Pentafluorosulfanyl)-1,4,4a,8a-tetrahydro-1,4-methanonaphthalene-5,8-dione (16).** Quinone **12** (0.150 g, 0.641 mmol) was dissolved in CH_2Cl_2 (1 mL), cooled to 0 °C with ice under nitrogen atmosphere. Freshly distilled cyclopentadiene (0.060 mL, 0.71 mmol) was added and the ice was allowed to melt gradually. After 1.5 h the solvent was evaporated and the residue was purified by column chromatography on silica gel in petroleum ether/CH_2Cl_2 (1:1, v/v). The product **16** was obtained as a yellow solid (0.176 g, 92%); R_f 0.34 (petroleum ether/CH_2Cl_2, 1:1); mp 51-55 °C; FTIR (film, $CHCl_3$) ν_{max} (cm^{-1}) 1689, 1609, 855, 844, 606, 576; ^{1}H NMR (500 MHz, $CDCl_3$): δ 1.47 (dtt, 1H, *J* = 9.0, 1.4, 0.6 Hz), 1.59 (ddd, 1H, *J* = 8.9, 1.8, 1.8 Hz), 3.35 (dd, 1H, *J* = 8.8, 4.0 Hz), 3.42 (dd, 1H, *J* = 8.8, 3.9 Hz), 3.53-3.57 (m,

1H), 3.57-3.61 (m, 1H), 6.12 (dd, 1H, *J* = 5.6, 2.8 Hz), 6.15 (dd, 1H, *J* = 5.6, 2.8 Hz), 7.03 (s, 1H); ^{13}C NMR (125.7 MHz, $CDCl_3$): δ 49.2, 49.3, 49.7, 50.2, 50.8, 135.7, 135.8, 142.1 (quin, *J* = 4.4 Hz), 160.2 (quin, *J* = 12.3 Hz), 188.8, 197.8; ^{19}F NMR (376 MHz, $CDCl_3$): δ 65.5 (dm, 4F, *J* = 152.5 Hz), 76.6-78.4 (m, 1F); HRMS (EI) *m*/*z* calcd for $C_{11}H_9F_5O_2S$ $[M]^+$ 300.0243, found 300.0244.

2-(3-Hydroxy-5-oxo-2,5-dihydrofuran-2-yl)-acetic acid (17). Muconolactone **3** (0.105 g, 0.392 mmol) was dissolved water (3 mL) and stirred at RT. After 40 h the solution was extracted with Et_2O (3 x 8 mL), the extract was washed with NaCl (aq) (1 mL), dried with $MgSO_4$ and evaporated to give **17** as a white solid (37 mg, 60% yield). ^{1}H NMR (400 MHz, DMSO-d_6): δ 2.42 (dd, 1H, *J* = 16.3, 8.8 Hz), 2.84 (dd, 1H, *J* = 16.3, 3.5 Hz), 4.93 (d, 1H, *J* = 1.0 Hz), 5.05 (ddd, 1H, *J* = 8.8, 3.5, 1.1 Hz), 12.72 (br s, 1H, OH); ^{13}C NMR (100 MHz, DMSO-d_6): δ 36.8, 75.1, 87.9, 170.6, 172. 7, 181.0; HRMS (ESI^-) *m*/*z* calcd for $C_6H_5O_5$ $[M - H]^-$ 157.0143, found 157.0140. ^{1}H NMR shifts are in agreement with literature values [1].

4-Oxo-3-(pentafluorosulfanyl)pentanoic acid (18). A solution of muconolactone **3** (0.253 g, 0.943 mmol) in H_3PO_4 (2.5 mL, 85 wt %) was heated in a 100 °C bath for 100 min. After cooling, the mixture was diluted with water (9 mL) and extracted with CH_2Cl_2 (3 × 15 mL). The organic extract was washed with water (4 mL), NaCl (aq) (4 mL), dried with $MgSO_4$ and evaporated to give 70 mg crude product. The crude product was dissolved in $NaHCO_3$ (aq) (8 mL, 2.5 wt %) and extracted with CH_2Cl_2 (5 mL). This extract was discarded. The aqueous solution was stirred for 15 min at RT, then acidified with HCl (aq, conc.) and extracted with CH_2Cl_2 (3 × 8 mL). The organic extract was washed with water (2 mL), NaCl (aq) (2 mL), dried with $MgSO_4$ and evaporated to give **18** as a white solid (33.1 mg, 14%); mp 99-102 °C; FTIR ($CHCl_3$) ν_{max} (cm^{-1}) 3506, 1735, 1715, 866, 852; ^{1}H NMR (400 MHz, acetone-d_6): δ 2.47 (s, 3H), 3.23 (dd, 1H, *J* = 17.2, 3.4 Hz), 3.41 (dd, 1H, *J* = 17.2, 11.4 Hz), 5.17-5.30 (m, 1H); ^{13}C NMR (100 MHz, acetone-d_6): δ 30.8-31.0 (m), 34.5 (quin, *J* = 4.4 Hz), 84.2 (quin, *J* = 8.6 Hz), 171.1-171.3 (m), 197.4-197.5 (m); ^{19}F NMR (376 MHz, acetone-d_6): δ 66.4 (dd, 4F, *J* = 143.3, 6.7 Hz), 83.2-84.9 (m, 1F); HRMS (CI) *m*/*z* calcd for $C_5H_8F_5O_3S$ $[M + H]^+$ 243.0114, found 243.0113.

(Pentafluorosulfanyl)maleic anhydride (20). (Pentafluorosulfanyl)maleic acid (0.225 g, 0.929 mmol) was heated in a Schlenk tube in a 125 °C bath at 25 Torr. On the cold parts of the tube white solid formed. The solid obtained was the anhydride **20** and weighed 0.173 g (83% yield); mp 52-54 °C; FTIR (solution in $CHCl_3$) ν_{max} (cm^{-1}) 3119, 1864, 1792, 1269, 1229, 951, 882, 851, 610, 577; ^{1}H NMR (400 MHz, $CDCl_3$): δ 7.35 (s, 1H); ^{13}C NMR (125.7 MHz, $CDCl_3$): δ 135.0 (quin, *J* = 4.6 Hz), 155.2 (quind, *J* = 25.5, 2.4 Hz), 155.9 (quin, *J* = 2.3 Hz), 158.9; ^{19}F NMR (376 MHz, $CDCl_3$): δ 67.3-67.9 (m, 4F), 70.4-72.1 (m); MS (EI) *m*/*z* (rel. int.) 53 (100), 70 (7), 89 (25), 127 (10), 133 (19), 161 (10), 180 (10), 224 (3); HRMS (CI) *m*/*z* calcd for $C_4H_2F_5O_3S$ $[M + H]^+$ 224.9645, found 224.9639.

2-(Pentafluorosulfanyl)-5-norbornene-2,3-dicarboxylic anhydride (21). To anhydride **20** (86.8 mg, 0.387 mmol) freshly distilled cyclopentadiene (0.3 mL, 3.6 mmol) was added, which dissolved the anhydride immediately. The solution was stirred under argon at RT. After 1 h 45 min the mixture was analyzed and NMR showed the disappearance of the starting material and the formation of two new SF_5 compounds. The reaction mixture was evaporated at 20 °C/3 Torr to give 126 mg semisolid material. Chromatography on silicagel, elution with hexane/Et_2O (2:1) gave 10 mg mixture of the cyclopentadiene adducts and 44 mg of mostly pure major isomer. The total yield for both isomers is 54 mg, 48%. The products were quickly decomposing even when stored in the fridge. *endo*-**(21)**: FTIR ($CHCl_3$) ν_{max} (cm^{-1}) 3032, 1871, 1793, 892, 879, 867, 858, 843, 817, 801, 595, 579; ^{1}H NMR (500 MHz, $CDCl_3$): δ 2.08 (d, 1H, *J* = 9.8 Hz), 2.17 (d, 1H, *J* = 9.8 Hz), 3.60-3.66 (m, 1H), 3.93-3.97 (m, 1H), 4.28 (d, 1H, *J* = 4.8 Hz), 6.35 (dd, 1H, *J* = 5.6, 3.4 Hz), 6.46 (dd, 1H, *J* = 5.6, 2.9 Hz); ^{13}C NMR (125.7 MHz, $CDCl_3$): *δ* 47.0, 52.3, 52.5 (quin, *J* = 2.9 Hz), 53.5 (quin, *J* = 3.7 Hz), 100.9 (quin, *J* = 9.5 Hz), 137.2, 139.2, 163.2, 167.3; ^{19}F NMR (376 MHz, $CDCl_3$): *δ* 62.57 (d, 4F, *J* = 145.7 Hz), 79.9-81.6 (m, 1F); HRMS (CI) *m*/*z* calcd for $C_9H_8F_5O_3S$ $[M + H]^+$ 291.0114, found 291.0120. *exo*-**(21)**: ^{1}H NMR (400 MHz, $CDCl_3$): *δ* 1.58 (d, 1H, *J* = 11.2 Hz), 1.84-1.90 (m, 1H), 3.47-3.51 (m, 1H), 3.54 (dd, 1H, *J* = 2.1, 0.9 Hz), 3.86-3.90 (m, 1H), 6.37-6.42 (m, 1H), 6.57 (dd, 1H, *J* = 5.7, 3.3 Hz); ^{19}F NMR (376 MHz, $CDCl_3$): *δ* 63.36 (d, 4F, *J* = 148.0 Hz), 79.9-81.5 (m, 1F).

Dimethyl 3-(pentafluorosulfanyl)-4,5-dihydro-3*H*-pyrazole-3,4-dicarboxylate (22). To acid **4** (50.7 mg, 0.209 mmol) in Et_2O (1 mL), diazomethane in Et_2O (15 mL, ≈2 mmol) was added. The solution was allowed to stand 1.5 h at RT. Then the solution was dried with $MgSO_4$ and the solvent was removed under vacuum giving a very lightly yellow solid (63.4 mg, 98%); mp 76-79 °C; FTIR ($CHCl_3$) ν_{max} (cm^{-1}) 2957, 1757, 1748, 1562, 1268, 892, 872, 829, 804, 599, 572; ^{1}H NMR (400 MHz, $CDCl_3$): δ 3.70 (s, 3H), 3.81 (dd, 1H, *J* = 8.1, 3.1 Hz), 3.91 (s, 3H), 4.85 (dd, 1H, *J* = 18.7, 8.1 Hz), 5.11 (dd, 1H, *J* = 18.7, 3.1 Hz); ^{13}C NMR (100 MHz, $CDCl_3$) *δ* 44.6 (quin, *J* = 2.9 Hz), 53.2, 54.5, 82.1, 125.6-126.0 (m), 163.0, 170.1; ^{19}F NMR (376 MHz, $CDCl_3$): δ 56.9 (d, 4F, *J* = 146.1), 75.2-76.9 (m, 1F); HRMS (ESI^+) *m*/*z* calcd for $C_7H_9F_5N_2NaO_4S$ $[M + Na]^+$ 335.0095, found 335.0096.

(*E*)-3-(Pentafluorosulfanyl)acrylic acid (23) [2]. Acid **4** (25.9 mg, 0.107 mmol) was dissolved in DMSO-d_6 (0.55 mL), heated to 65 °C for 1.5 hours. Then internal standard 1-nitro-4-(pentafluorosulfanyl)benzene (35.7 mg, 0.143 mmol) was added. The NMR yield of **23** was 60%; ^{1}H NMR (400 MHz, DMSO-d_6): δ 6.82 (d, 1H, *J* = 14.6 Hz), 7.77 (dquin, 1H, *J* = 14.7, 7.0 Hz); ^{13}C NMR (100 MHz, DMSO-d_6) *δ* 129.8 (quin, *J* = 7.0 Hz), 151.2 (quin, *J* = 19.7 Hz), 164.1; ^{19}F NMR (376 MHz, DMSO-d_6) *δ* 64.5 (dd, 4F, *J* = 152.1, 7.2 Hz), 82.8-84.6 (m, 1F); MS (ESI^-) *m*/*z* 127, 197. HRMS (ESI^-) *m*/*z* calcd for $C_3H_2F_5O_2S$ $[M - H]^-$ 196.9701, found 196.9698.

(*E*)-3-[^{2}H]-3-(Pentafluorosulfanyl)acrylic acid, deutero-(23). Acid **4** (25 mg, 0.103 mmol) was twice coevaporated with D_2O (0.1 mL) at 30 °C/4 Torr, then with 1 mL

benzene at 35 °C/4 Torr. The dry residue was dissolved in DMSO-d_6 (0.6 mL), heated to 60 °C for 1.5 hours. NMR analysis showed the deuterated SF_5-acrylic acid (89% deuterium enrichment by ^1H NMR). ^1H NMR (500 MHz, DMSO-d_6): δ 6.82 s; ^{13}C NMR (125.7 MHz, DMSO-d_6) δ 129.7 (quin, J = 6.9 Hz), 151.4 (tquin, J = 28.3, 19.7 Hz), 164.2; ^{19}F NMR (376 MHz, DMSO-d_6) δ 64.4 (d, J = 152.2 Hz), 82.8-84.5 (m); HRMS (ESI$^-$) m/z calcd for $C_3H^2HF_5O_2S$ [M − H]$^-$ 197.9764, found 197.9763.

3. Copies of NMR spectra

(Pentafluorosulfanyl)benzoquinone (12), ^{1}H NMR (400 MHz, $CDCl_3$)

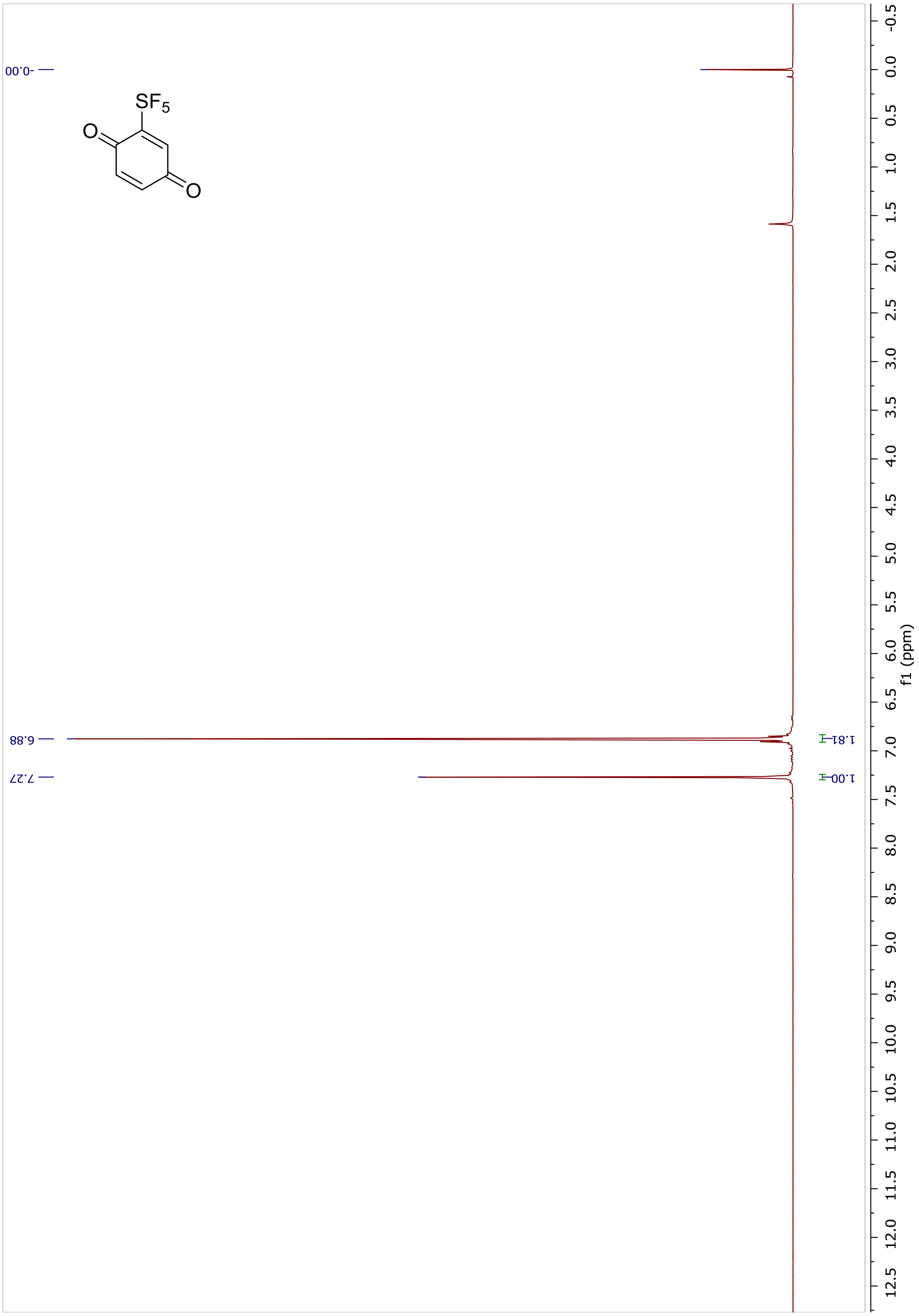

(Pentafluorosulfanyl)benzoquinone (12), ^{13}C NMR (100 MHz, $CDCl_3$)

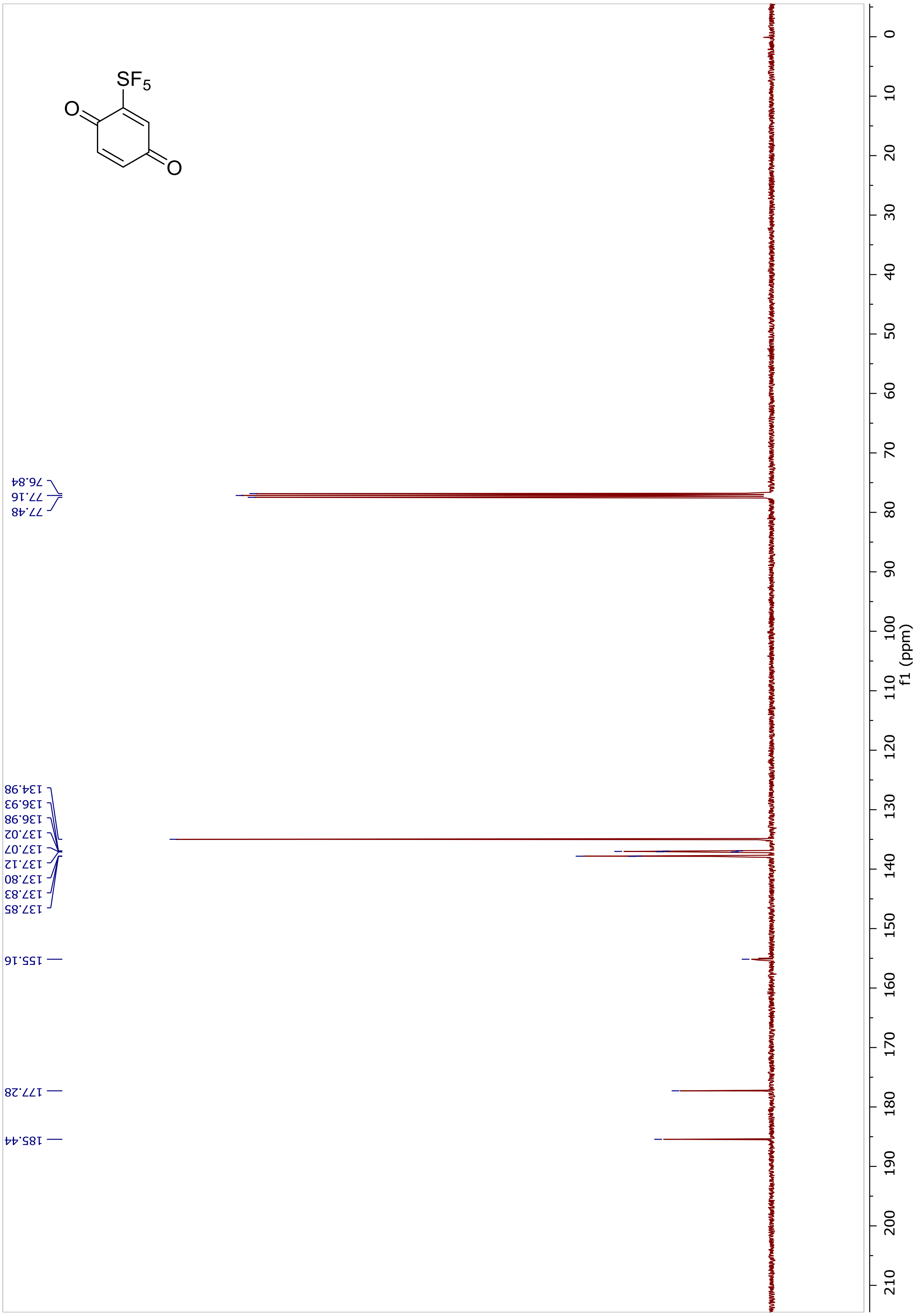

(Pentafluorosulfanyl)benzoquinone (12), ^{19}F NMR (376 MHz, $CDCl_3$)

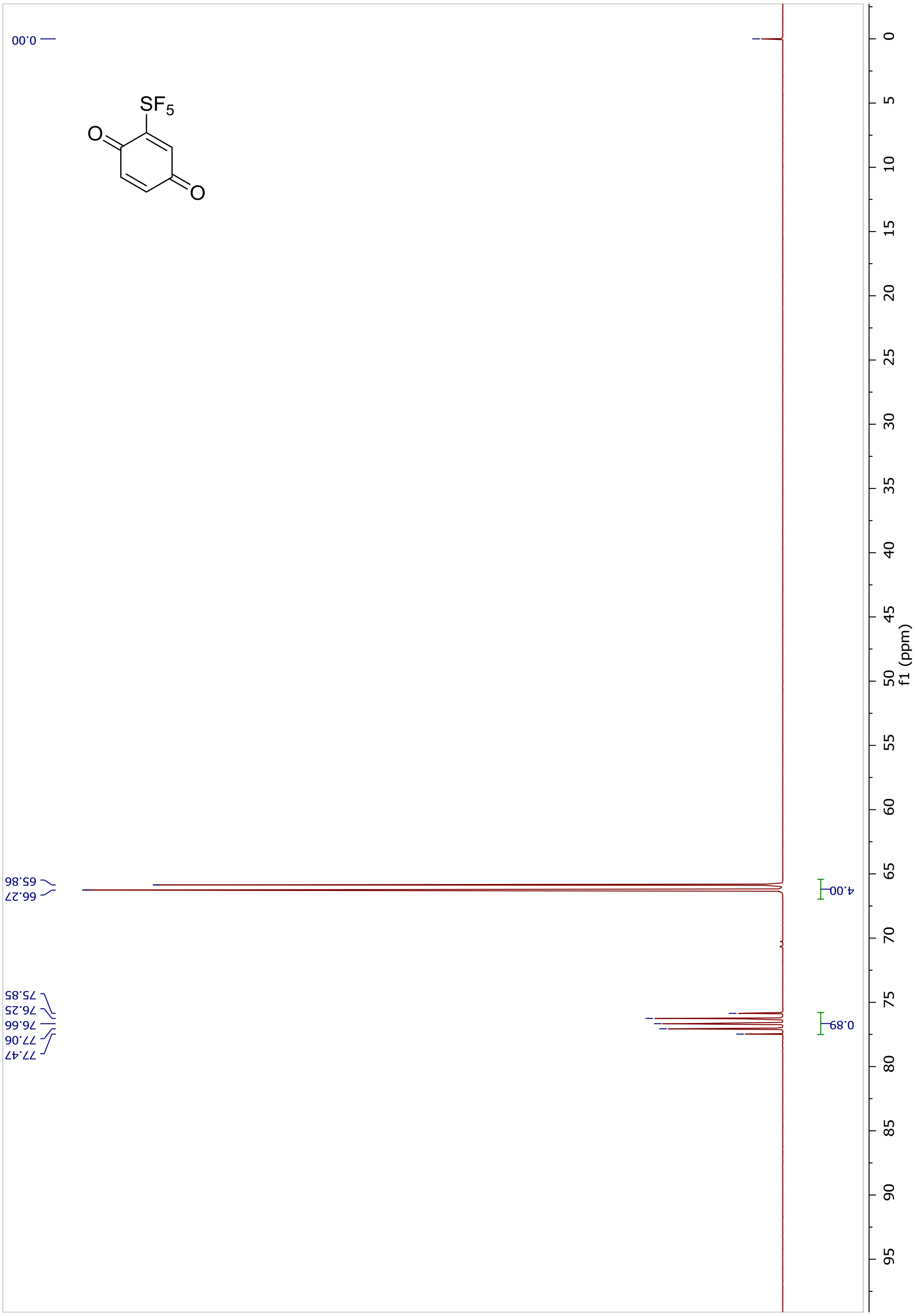

4-Amino-3-(pentafluorosulfanyl)phenol (14), ^{1}H NMR (400 MHz, DMSO-d_6)

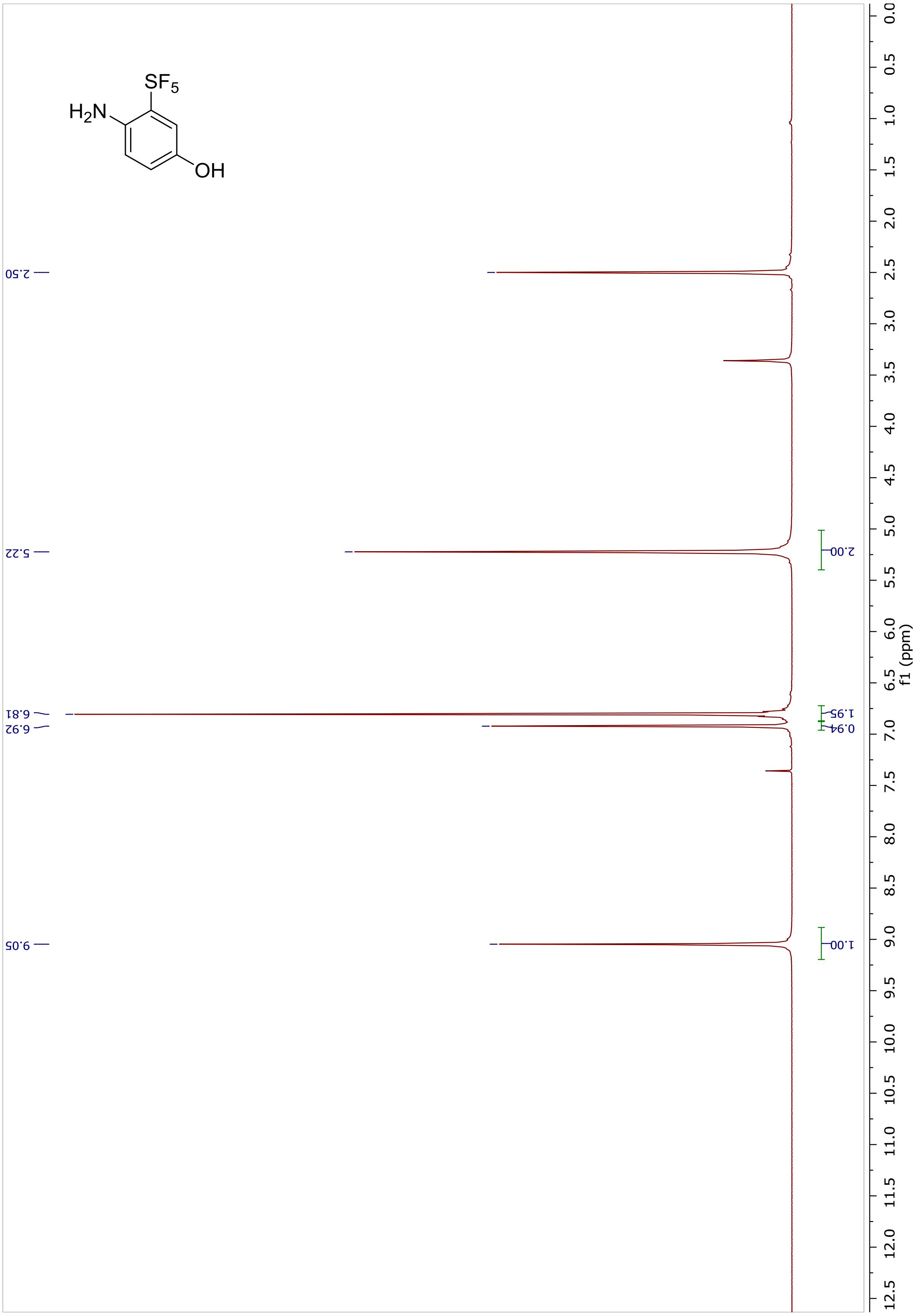

4-Amino-3-(pentafluorosulfanyl)phenol (14), ^{13}C NMR (100 MHz, DMSO-d_6), APT

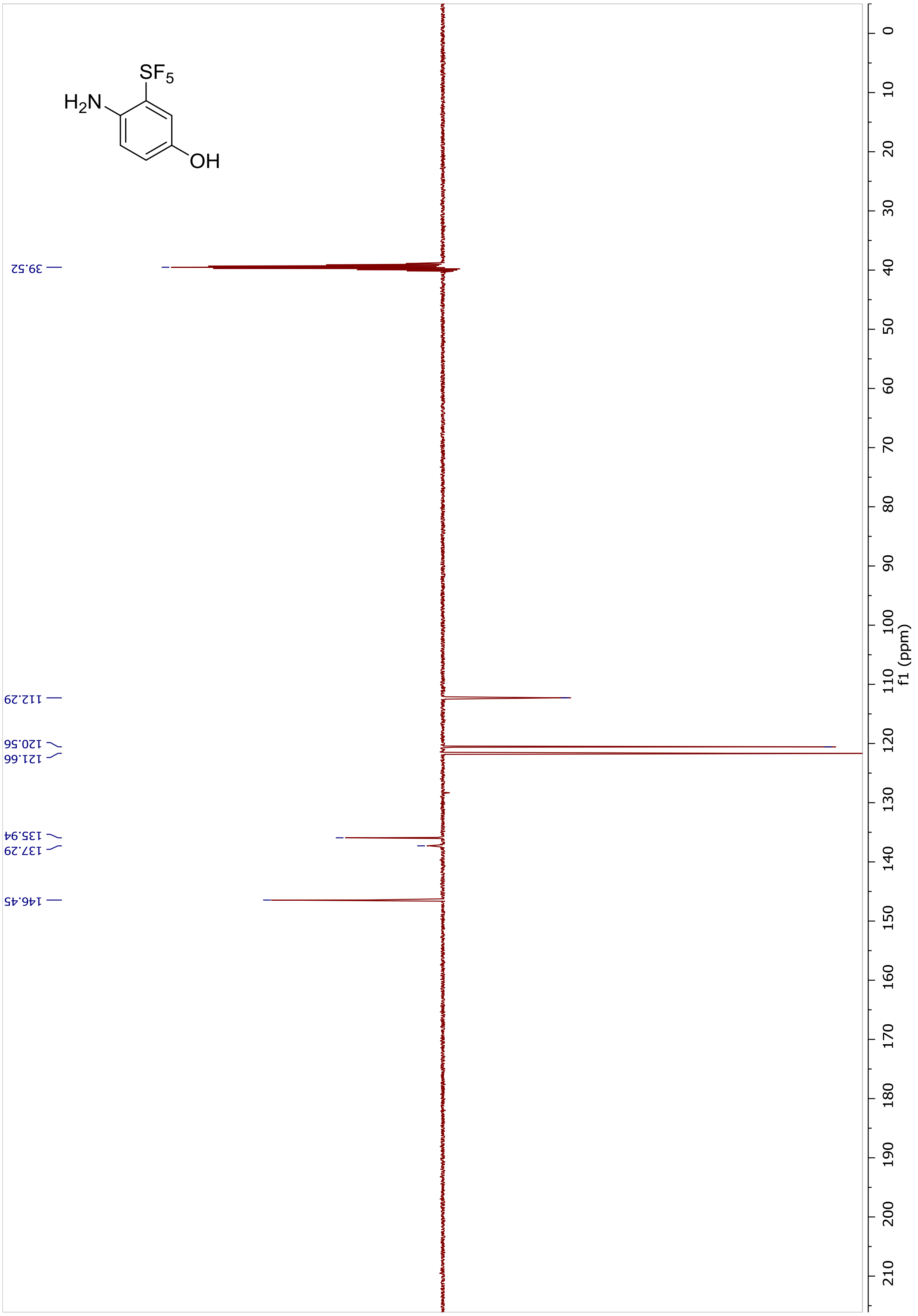

4-Amino-3-(pentafluorosulfanyl)phenol (14), ^{19}F NMR (376 MHz, DMSO-d_6)

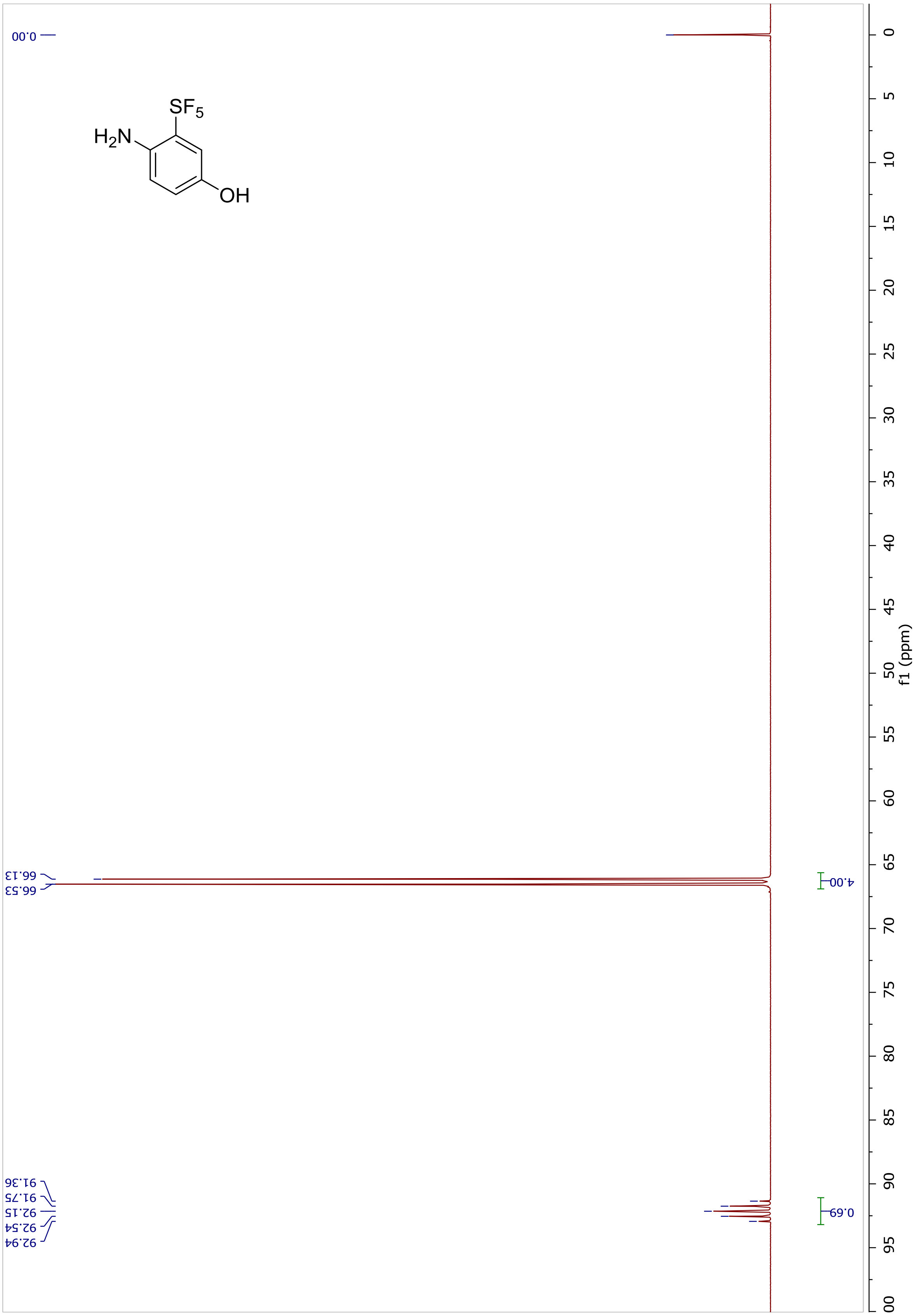

(Pentafluorosulfanyl)hydroquinone (15), ^{1}H NMR (400 MHz, acetone-d_6)

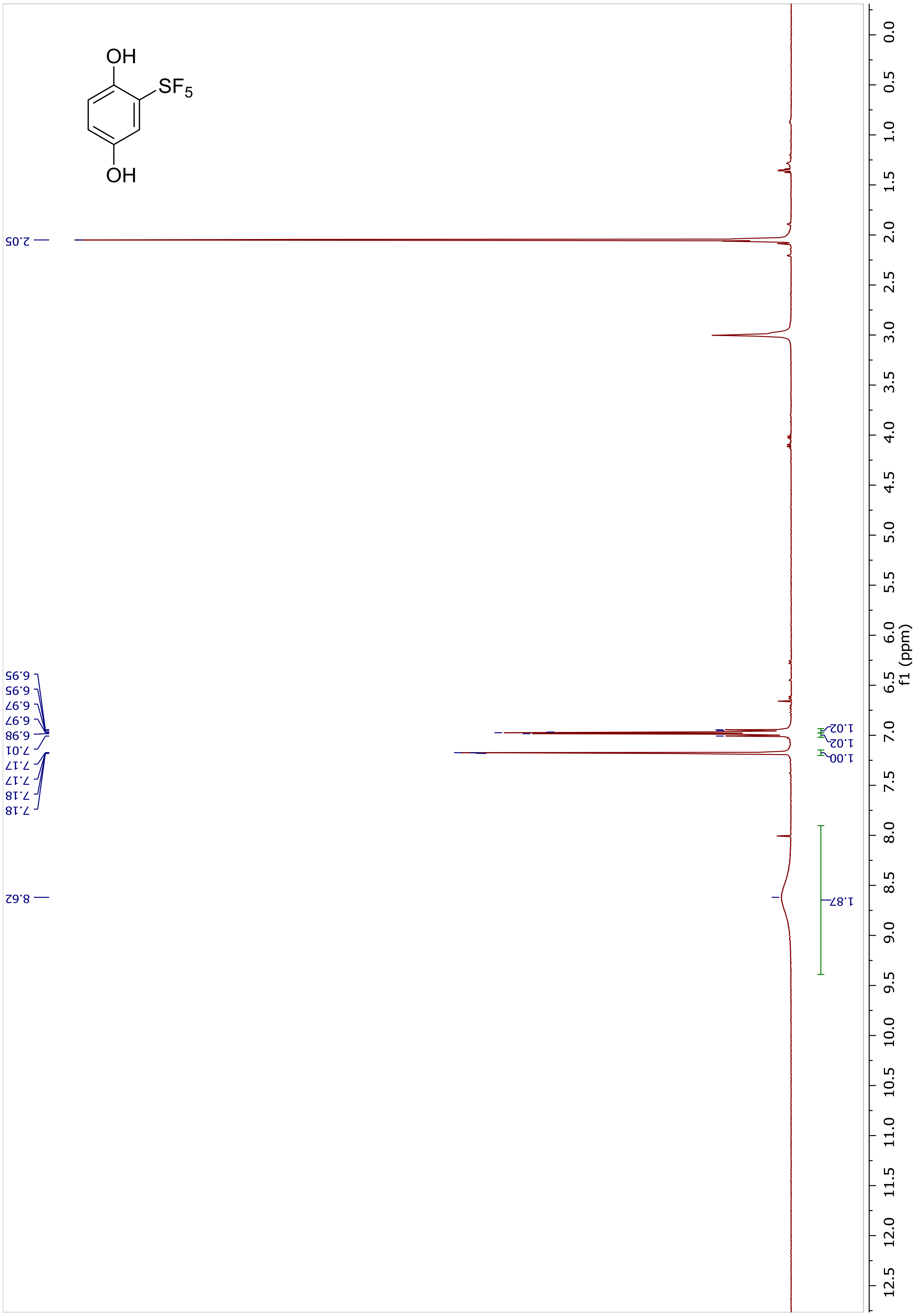

(Pentafluorosulfanyl)hydroquinone (15), ^{13}C NMR (100 MHz, acetone-d_6)

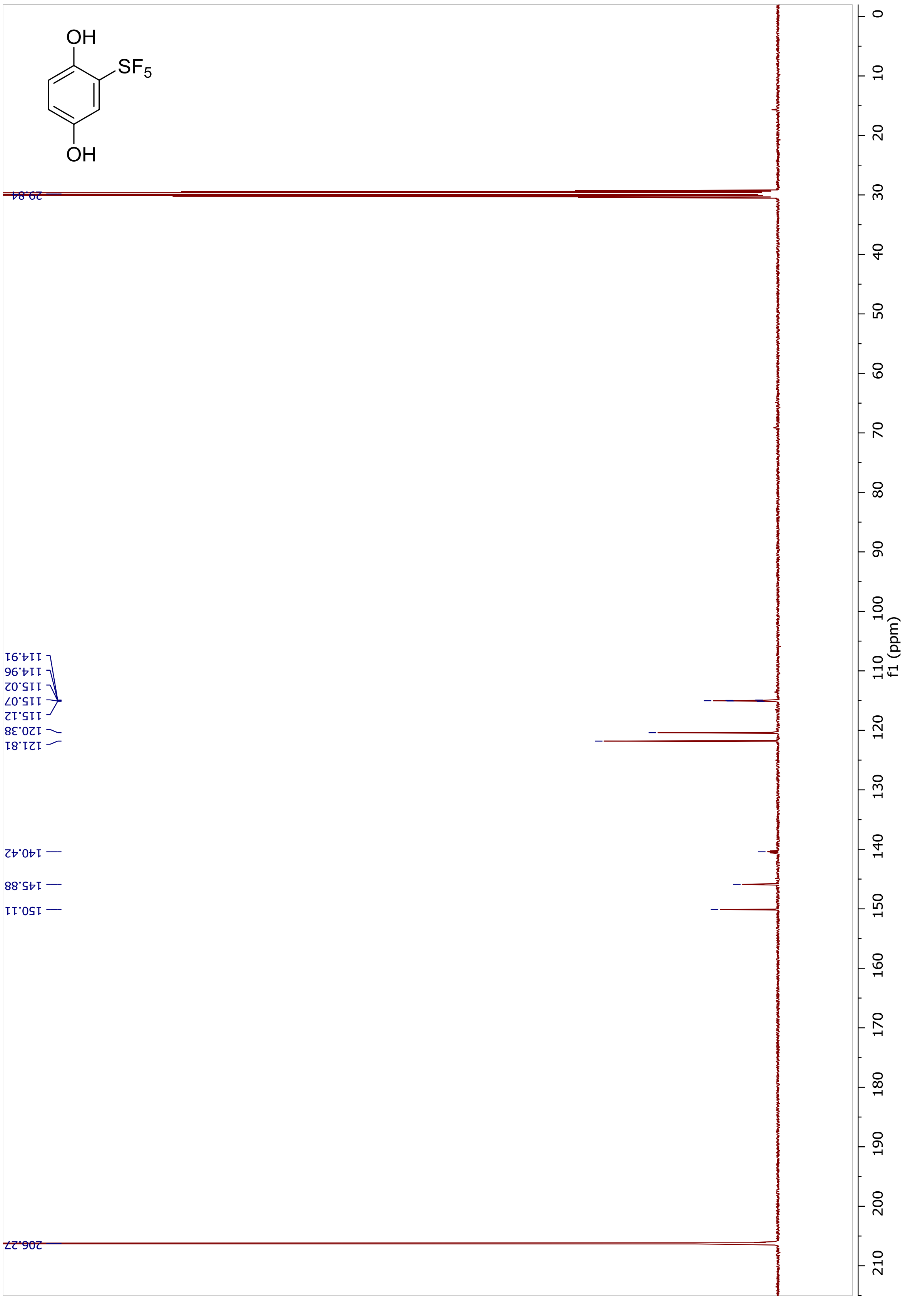

(Pentafluorosulfanyl)hydroquinone (15), ^{19}F NMR (376 MHz, acetone-d_6)

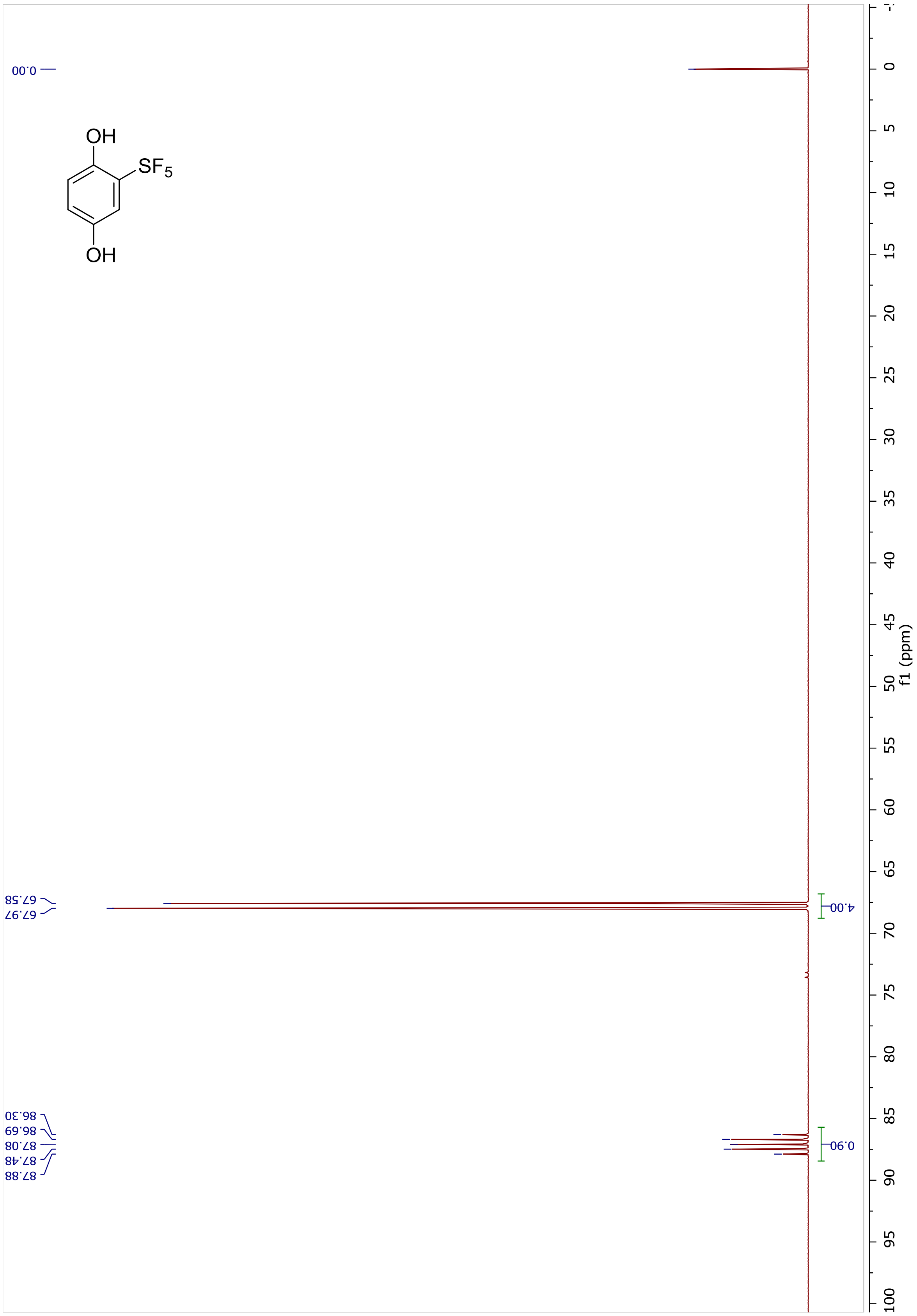

***endo*-6-(Pentafluorosulfanyl)-1,4,4a,8a-tetrahydro-1,4-methanonaphthalene-5,8-dione (16)**, ^{1}H NMR (500 MHz, $CDCl_3$)

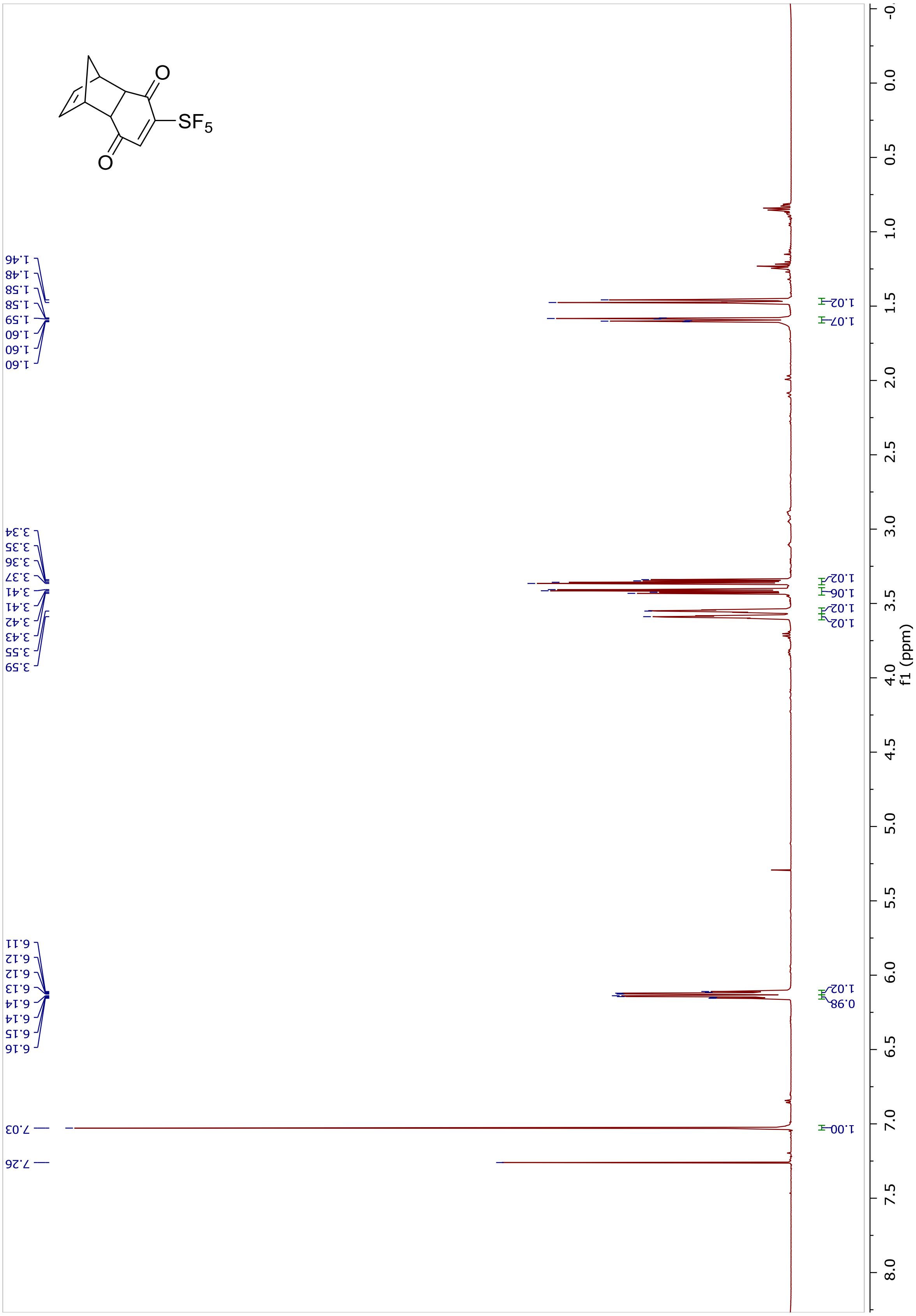

***endo*-6-(Pentafluorosulfanyl)-1,4,4a,8a-tetrahydro-1,4-methanonaphthalene-5,8-dione (16)**, ^{13}C NMR (125.7 MHz, $CDCl_3$), APT

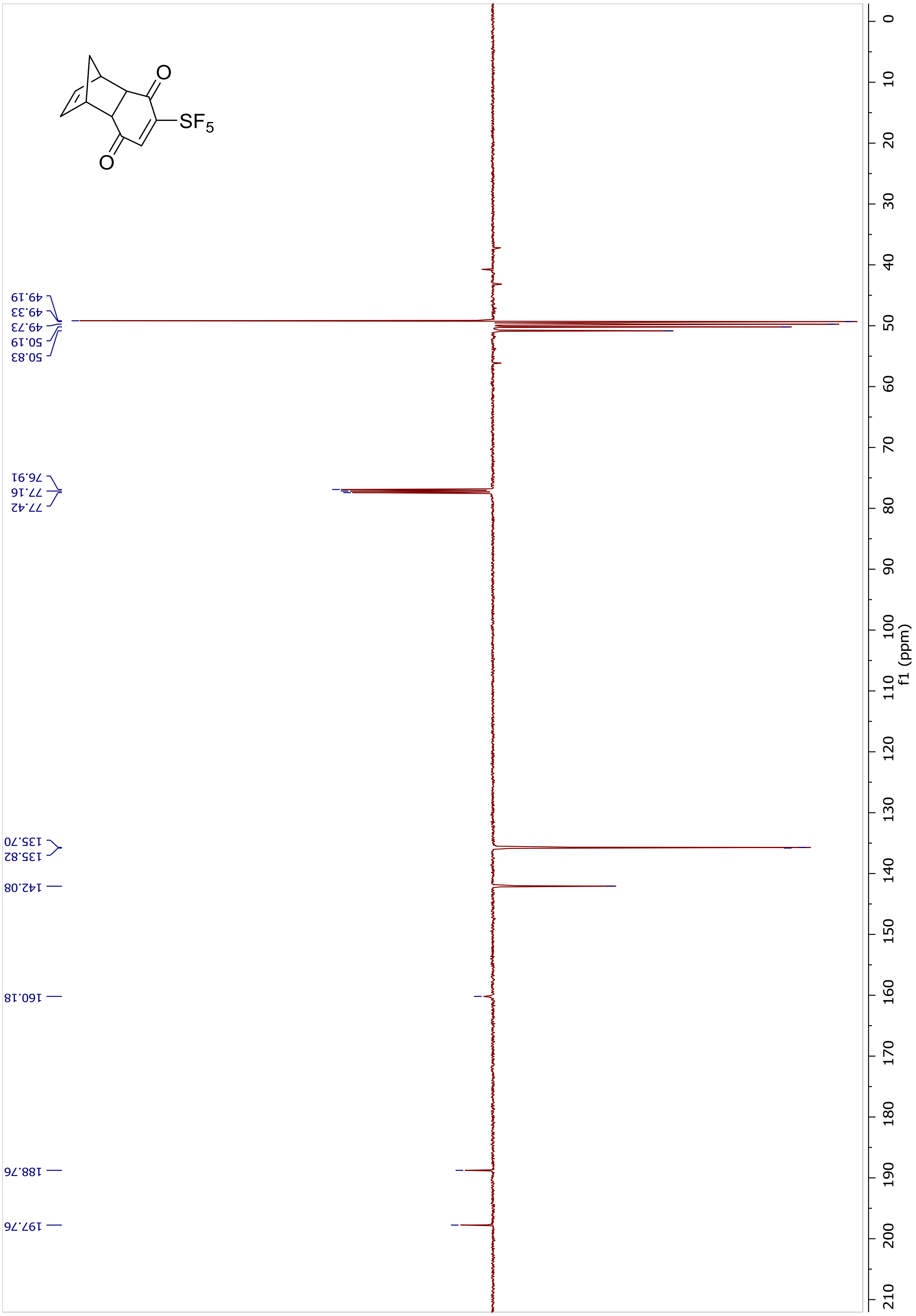

***endo*-6-(Pentafluorosulfanyl)-1,4,4a,8a-tetrahydro-1,4-methanonaphthalene-5,8-dione (16)**, ^{19}F NMR (376 MHz, $CDCl_3$)

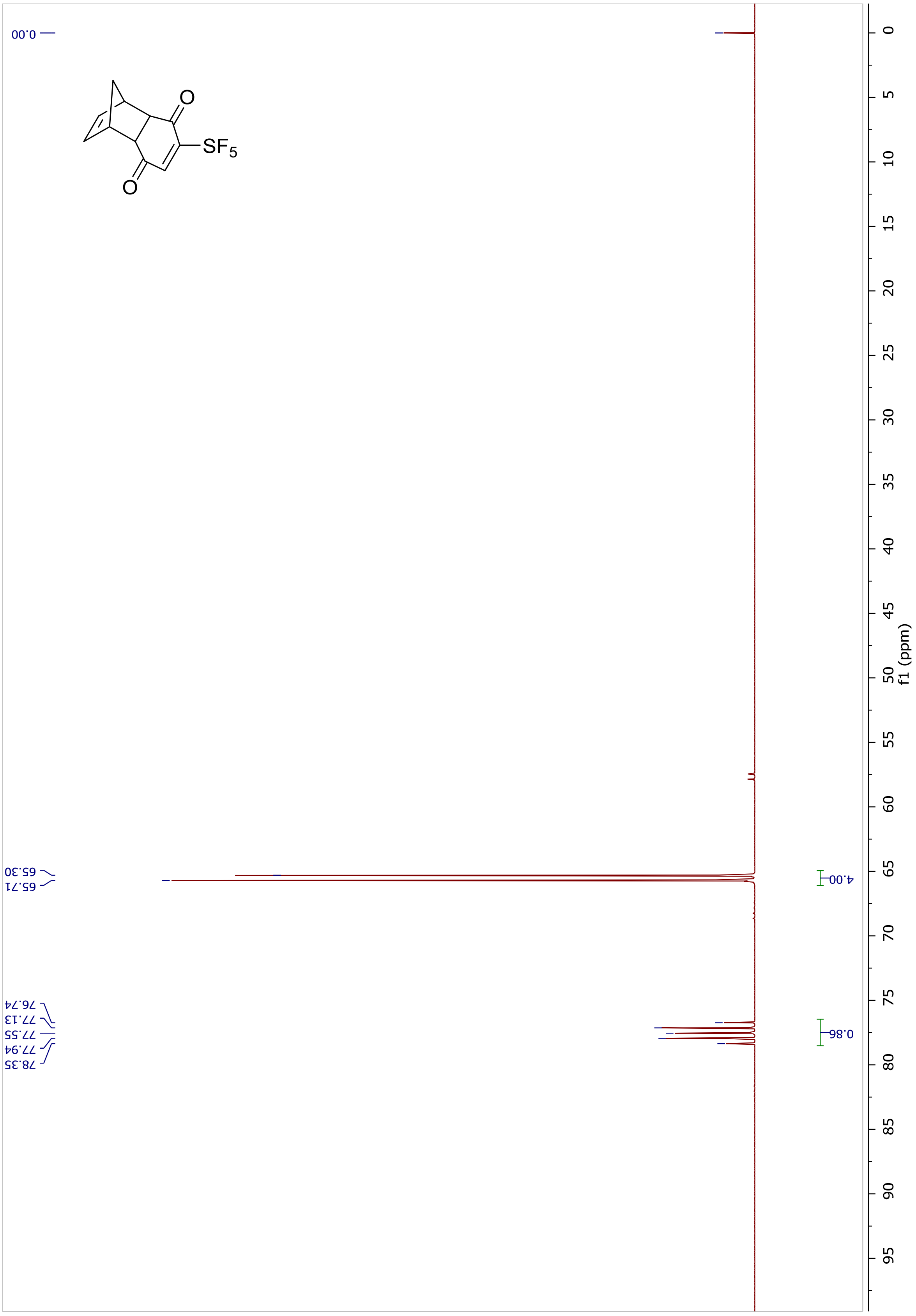

4-Oxo-3-(pentafluorosulfanyl)pentanoic acid (18), ^{1}H NMR (400 MHz, acetone-d_6)

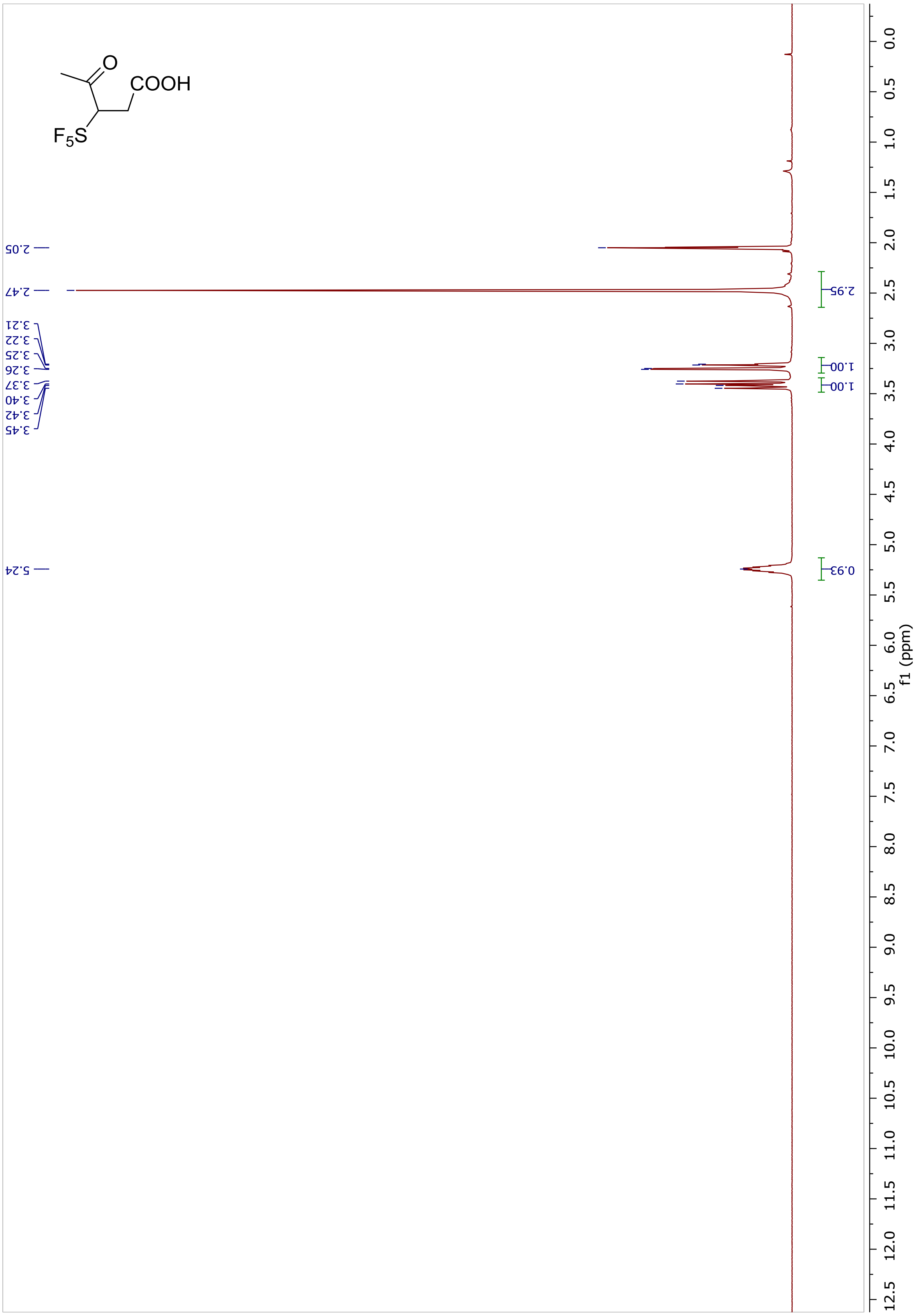

4-Oxo-3-(pentafluorosulfanyl)pentanoic acid (18), ^{13}C NMR (100 MHz, acetone-d_6)

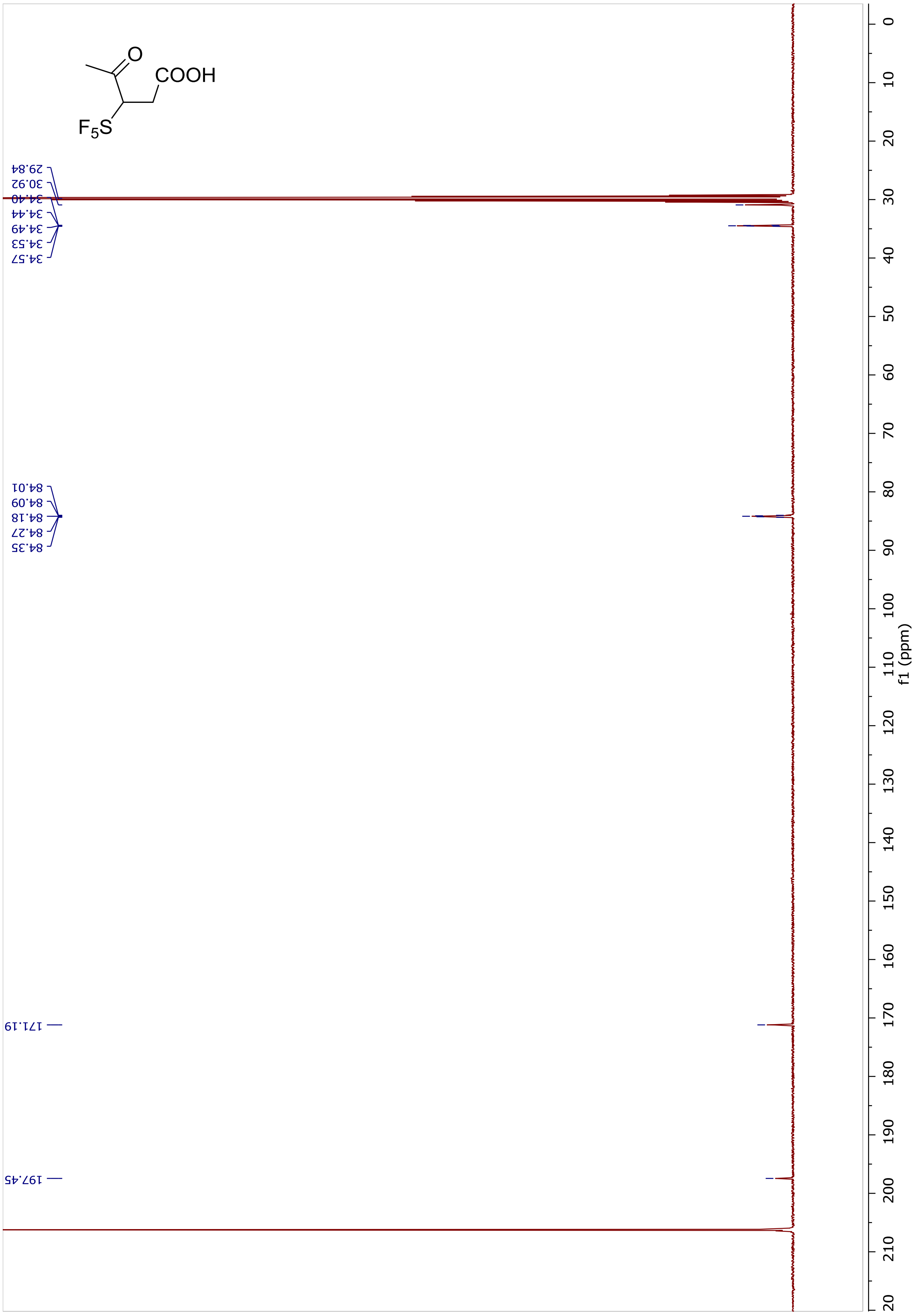

4-Oxo-3-(pentafluorosulfanyl)pentanoic acid (18), ^{19}F NMR (376 MHz, acetone-*d*$_6$)

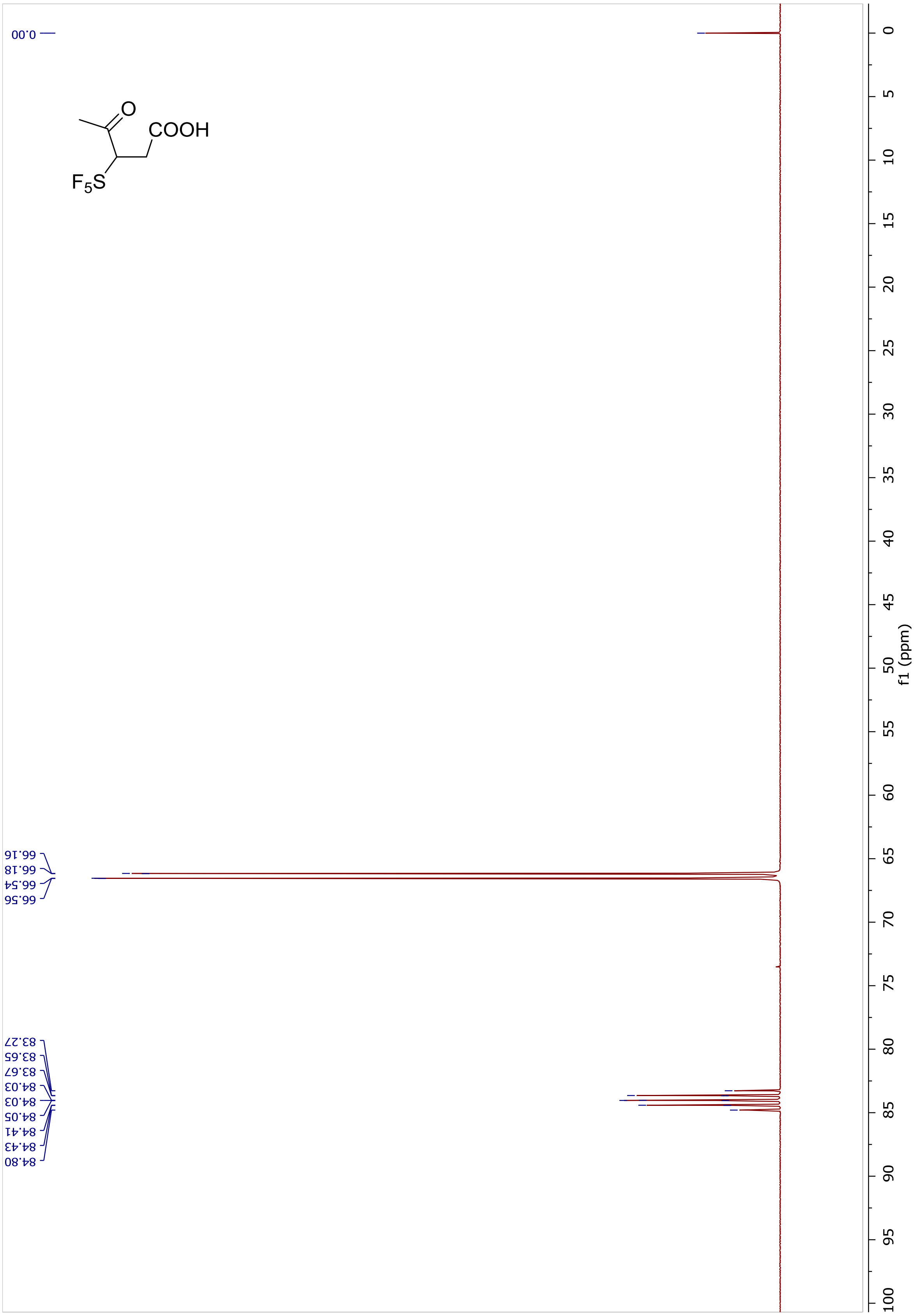

(Pentafluorosulfanyl)maleic anhydride (20), ^{1}H NMR (400 MHz, $CDCl_3$)

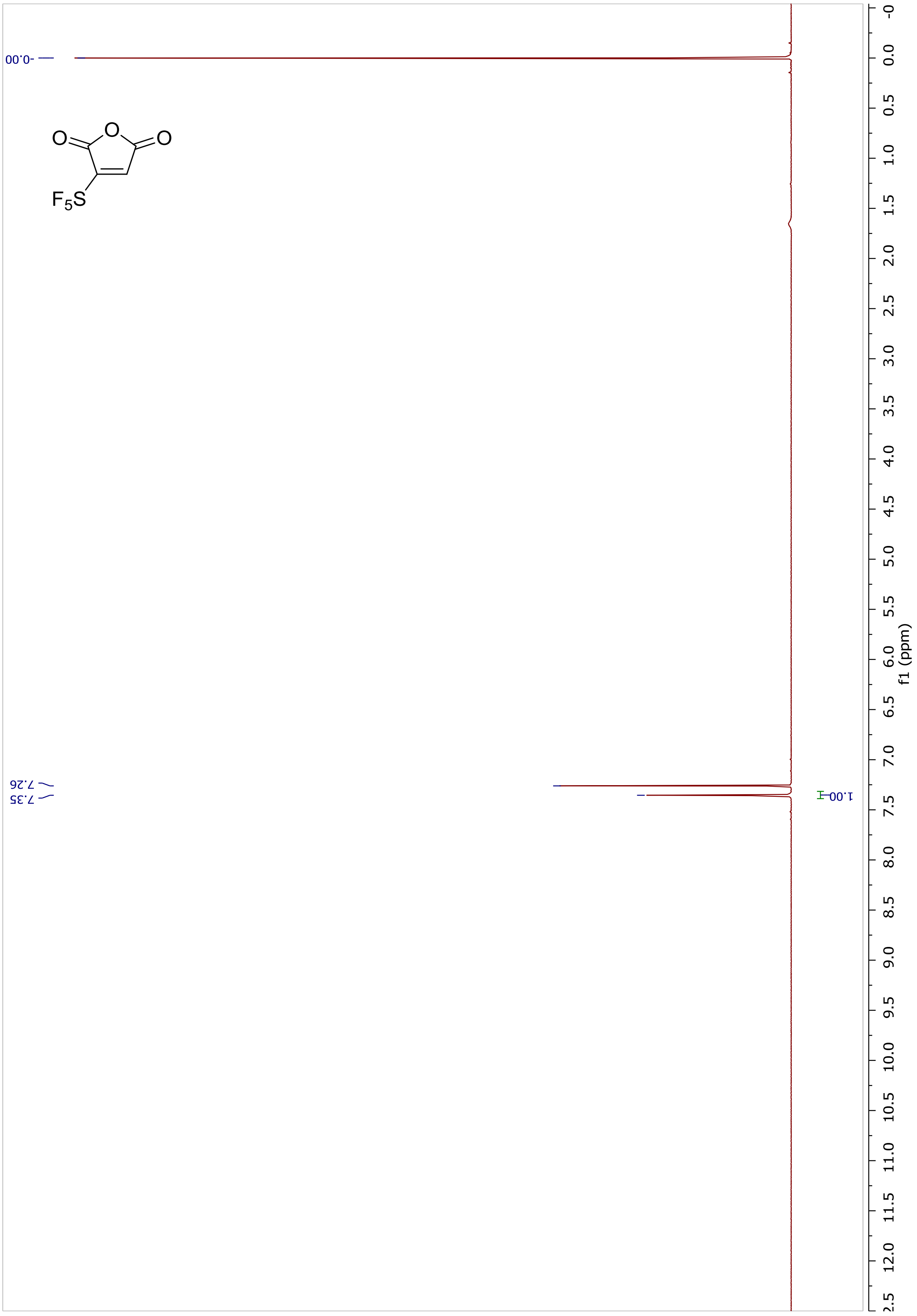

(Pentafluorosulfanyl)maleic anhydride (20), ^{13}C NMR (125.7 MHz, $CDCl_3$)

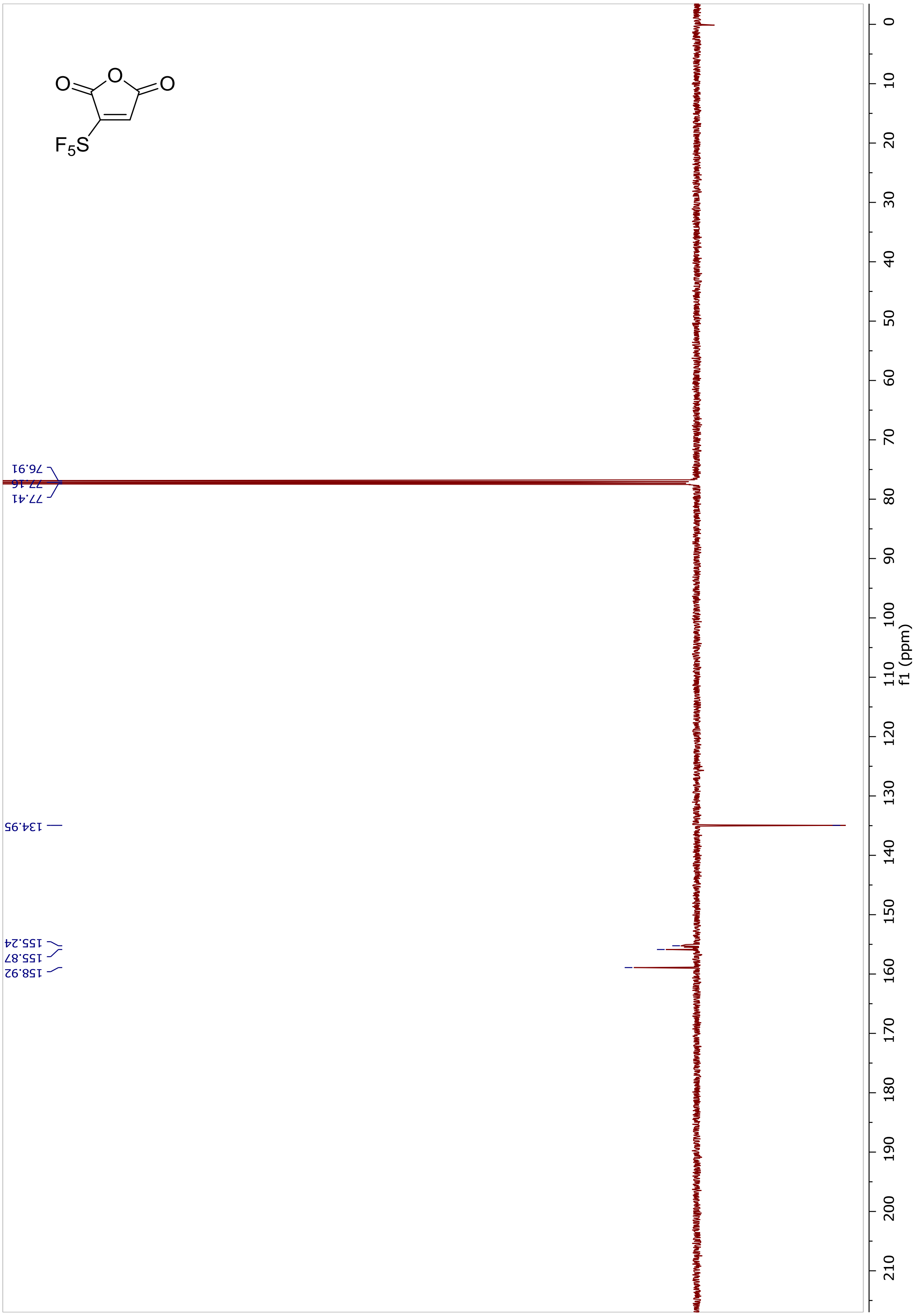

(Pentafluorosulfanyl)maleic anhydride (20), ^{19}F NMR (376 MHz, $CDCl_3$)

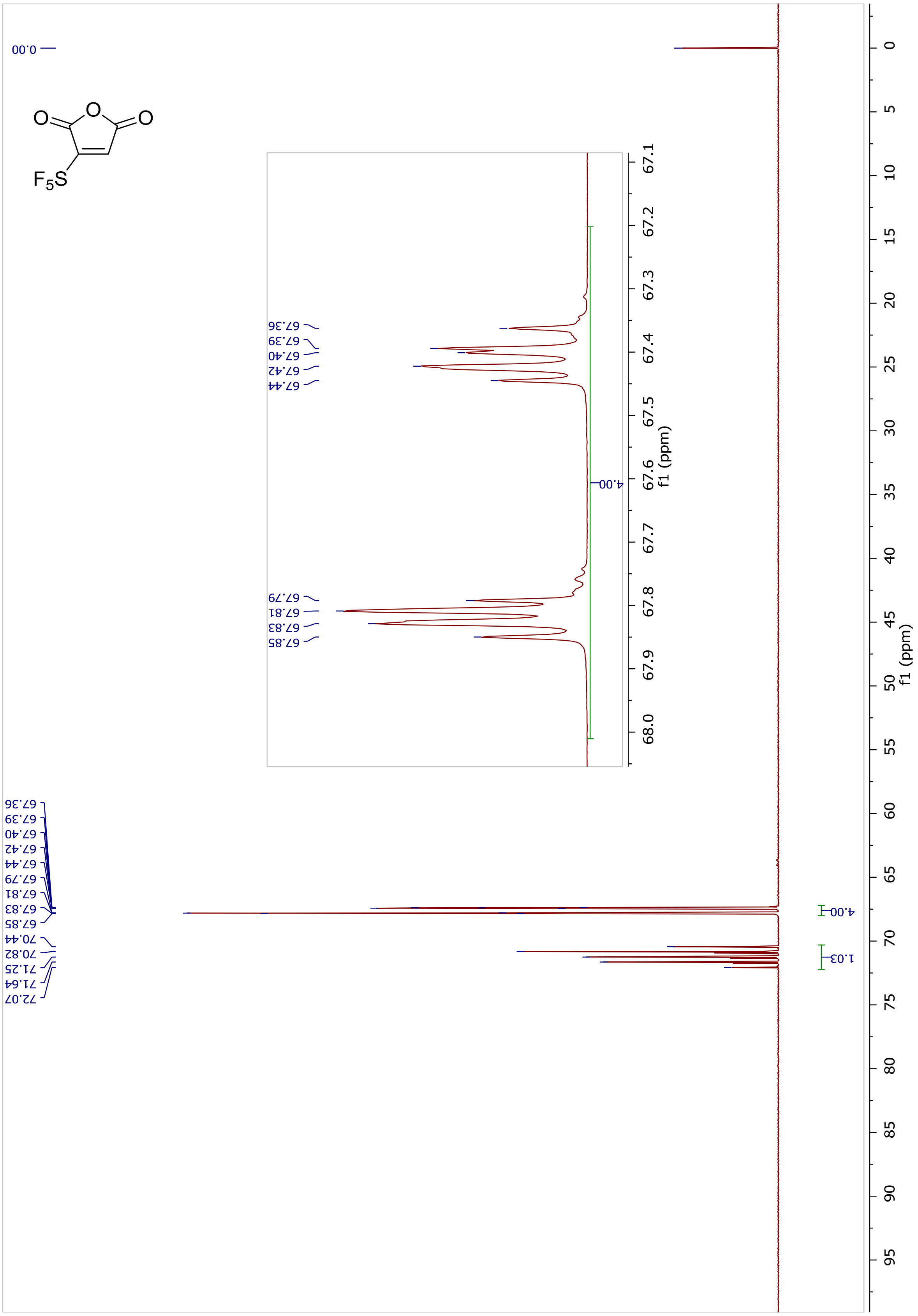

***endo*-2-(Pentafluorosulfanyl)-5-norbornene-2,3-dicarboxylic anhydride *endo*-(21)**, ^{1}H NMR (500 MHz, $CDCl_3$)

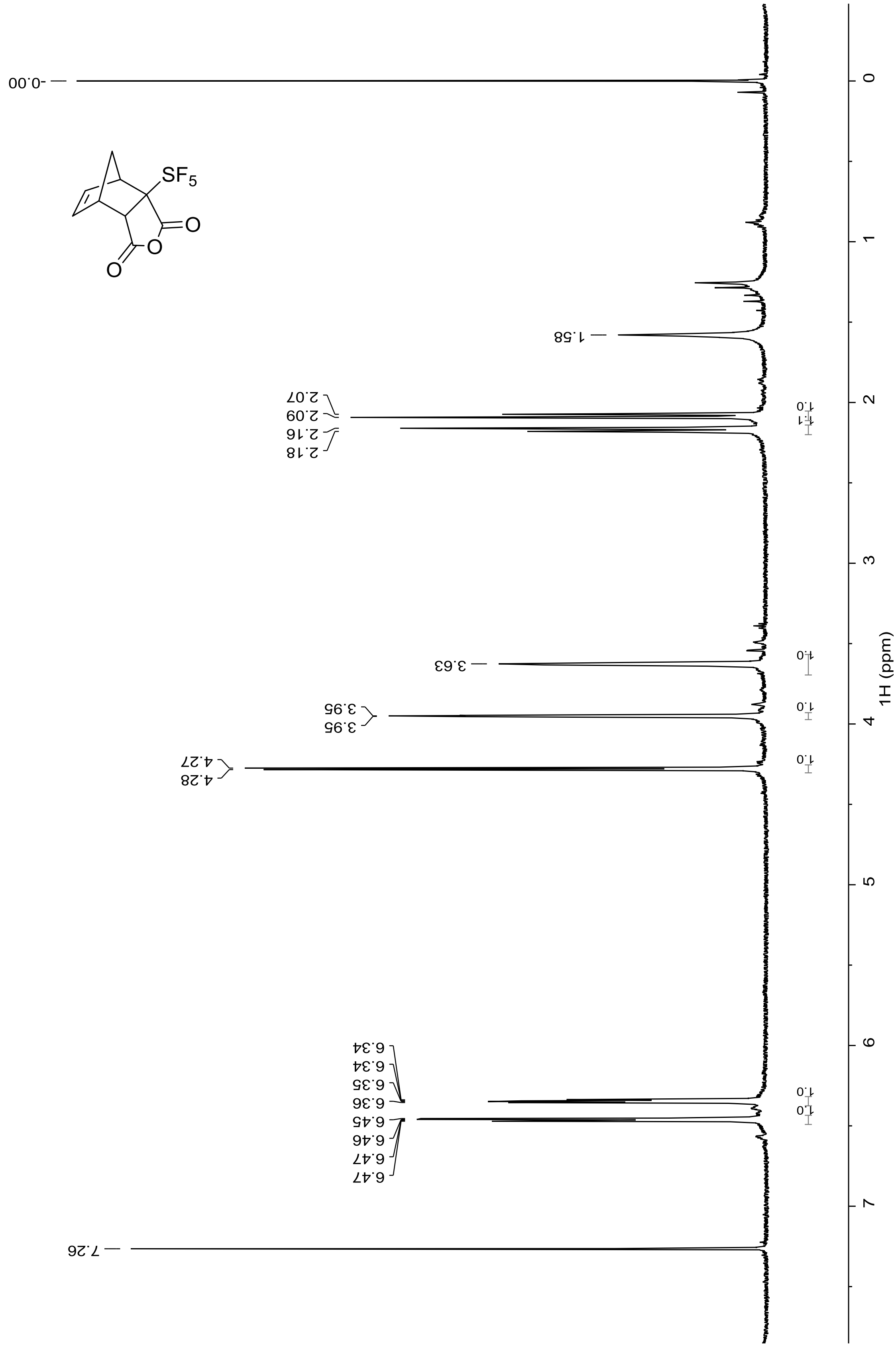

***endo*-2-(Pentafluorosulfanyl)-5-norbornene-2,3-dicarboxylic anhydride *endo*-(21)**, ^{13}C NMR (125.7 MHz, $CDCl_3$), APT

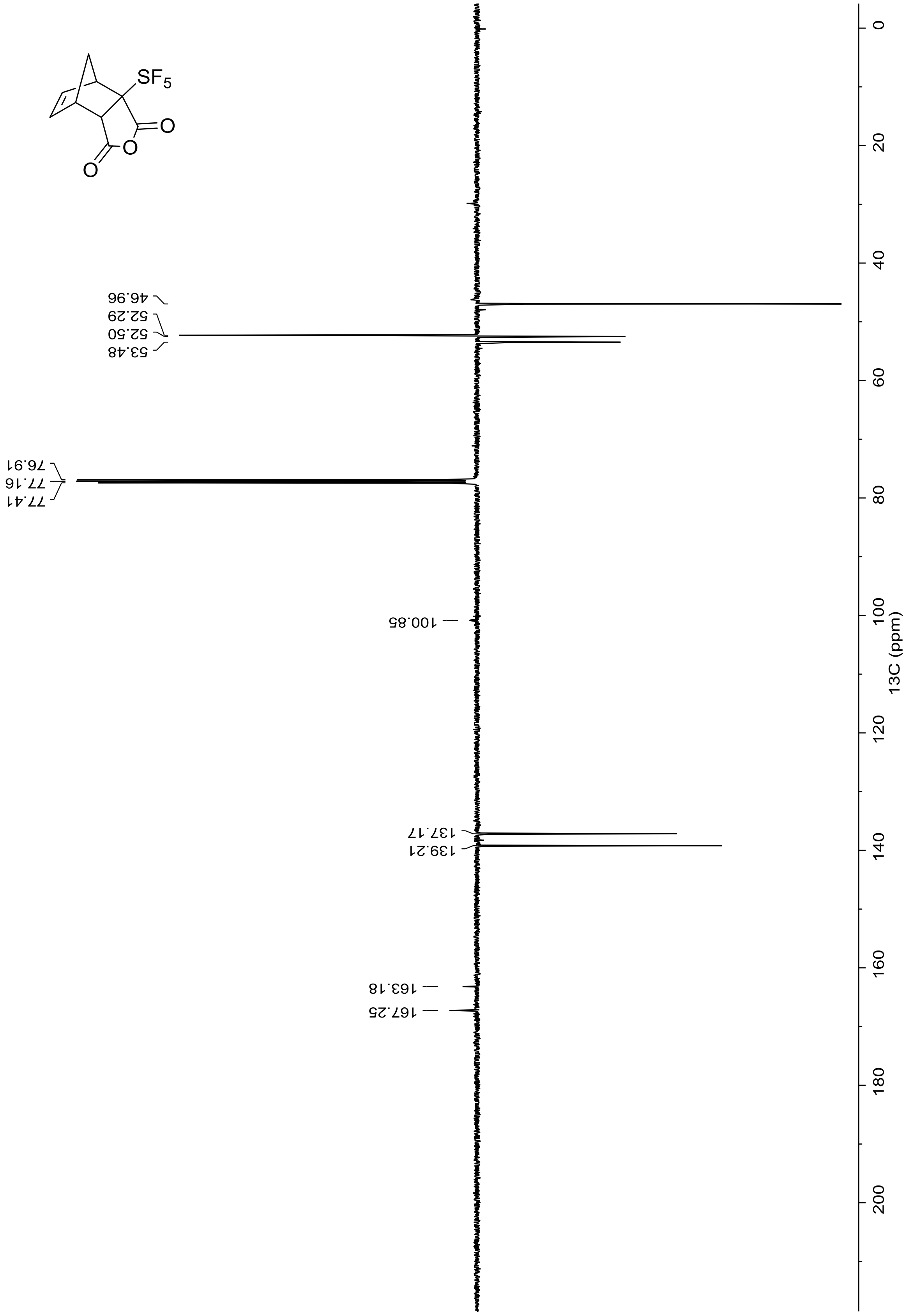

***endo*-2-(Pentafluorosulfanyl)-5-norbornene-2,3-dicarboxylic anhydride *endo*-(21)**, ^{19}F NMR (376 MHz, $CDCl_3$)

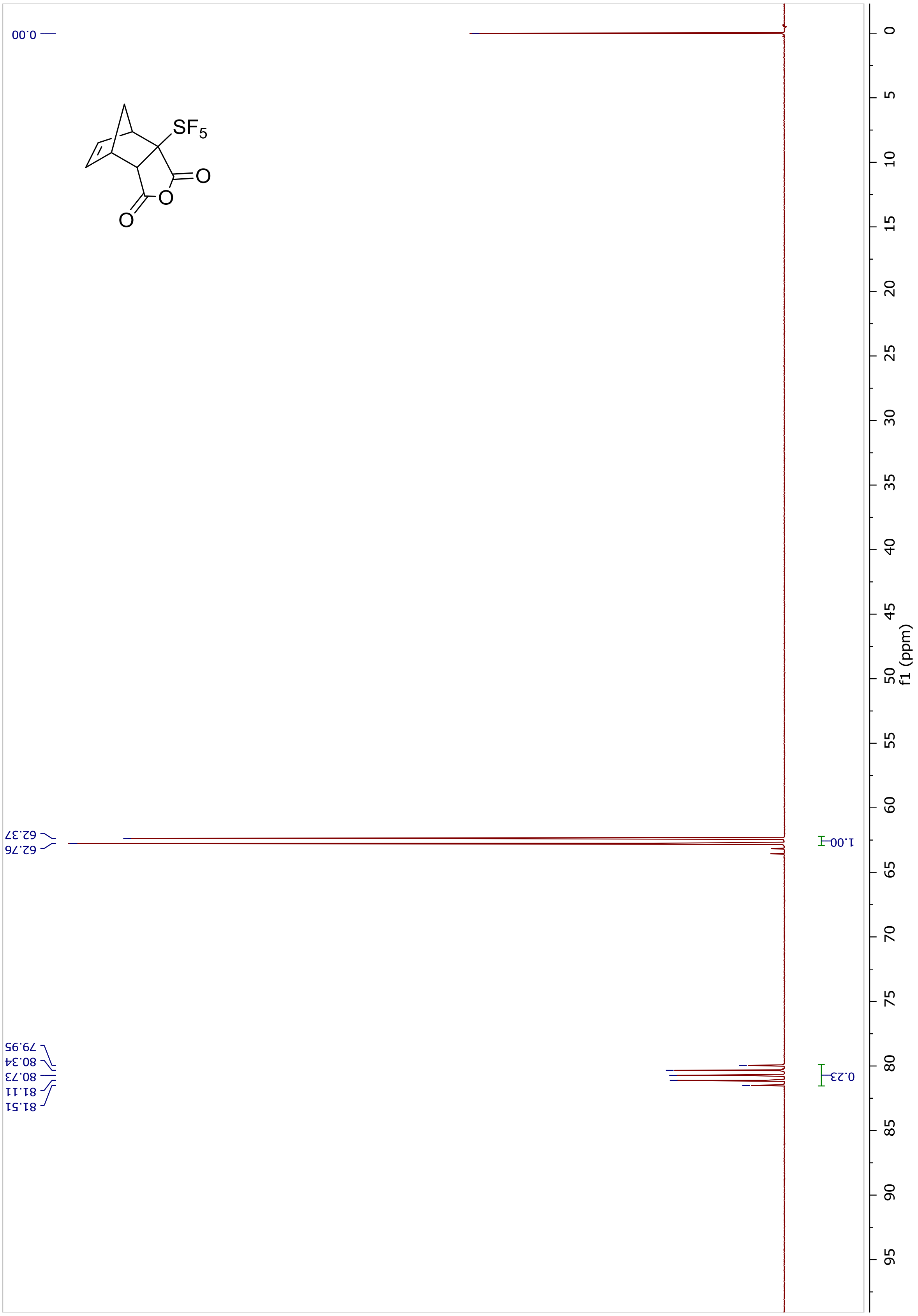

Dimethyl 3-(pentafluorosulfanyl)-4,5-dihydro-3*H*-pyrazole-3,4-dicarboxylate (22), ^{1}H NMR (400 MHz, $CDCl_3$)

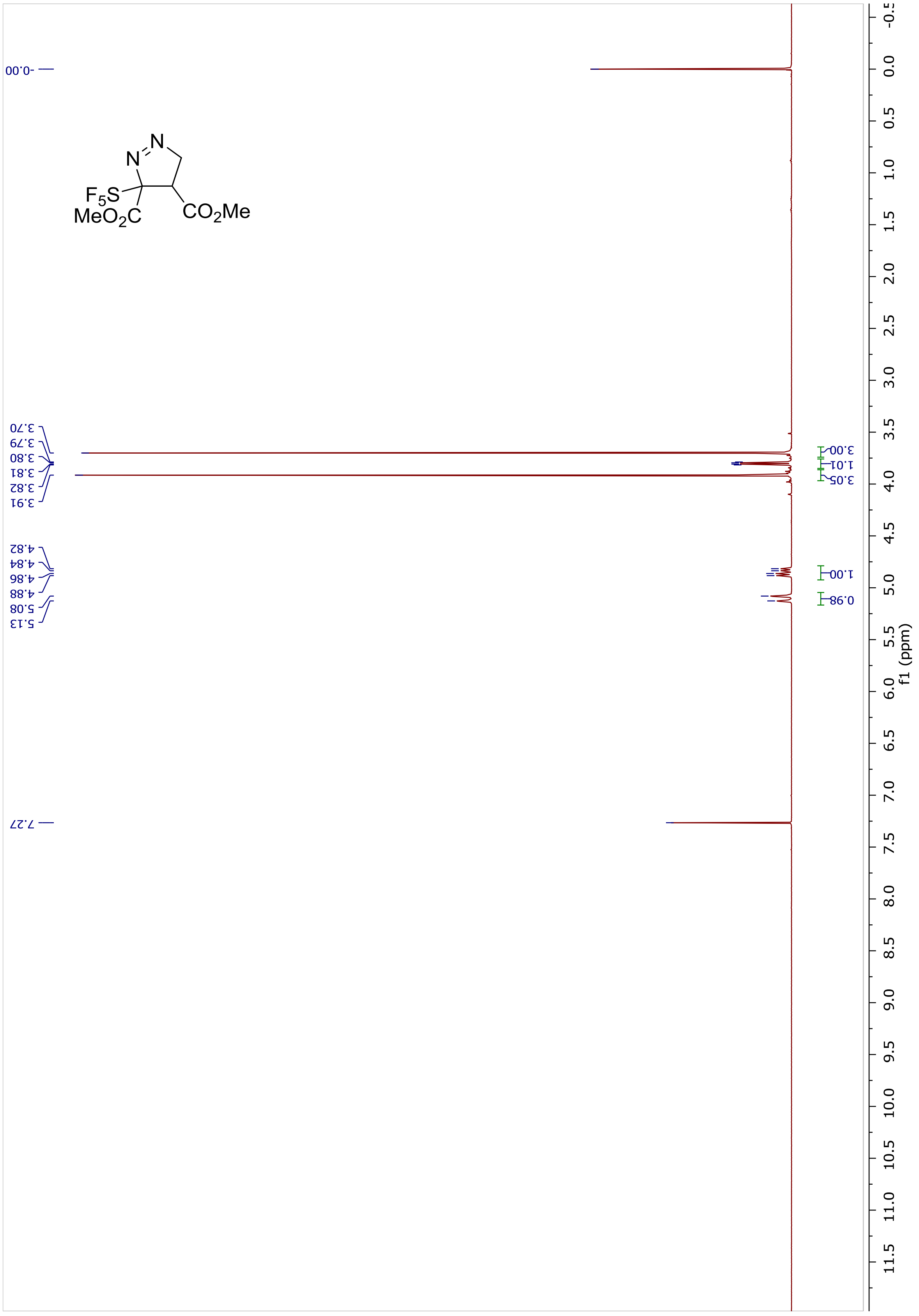

Dimethyl 3-(pentafluorosulfanyl)-4,5-dihydro-3*H*-pyrazole-3,4-dicarboxylate (22), ^{13}C NMR (100 MHz, $CDCl_3$)

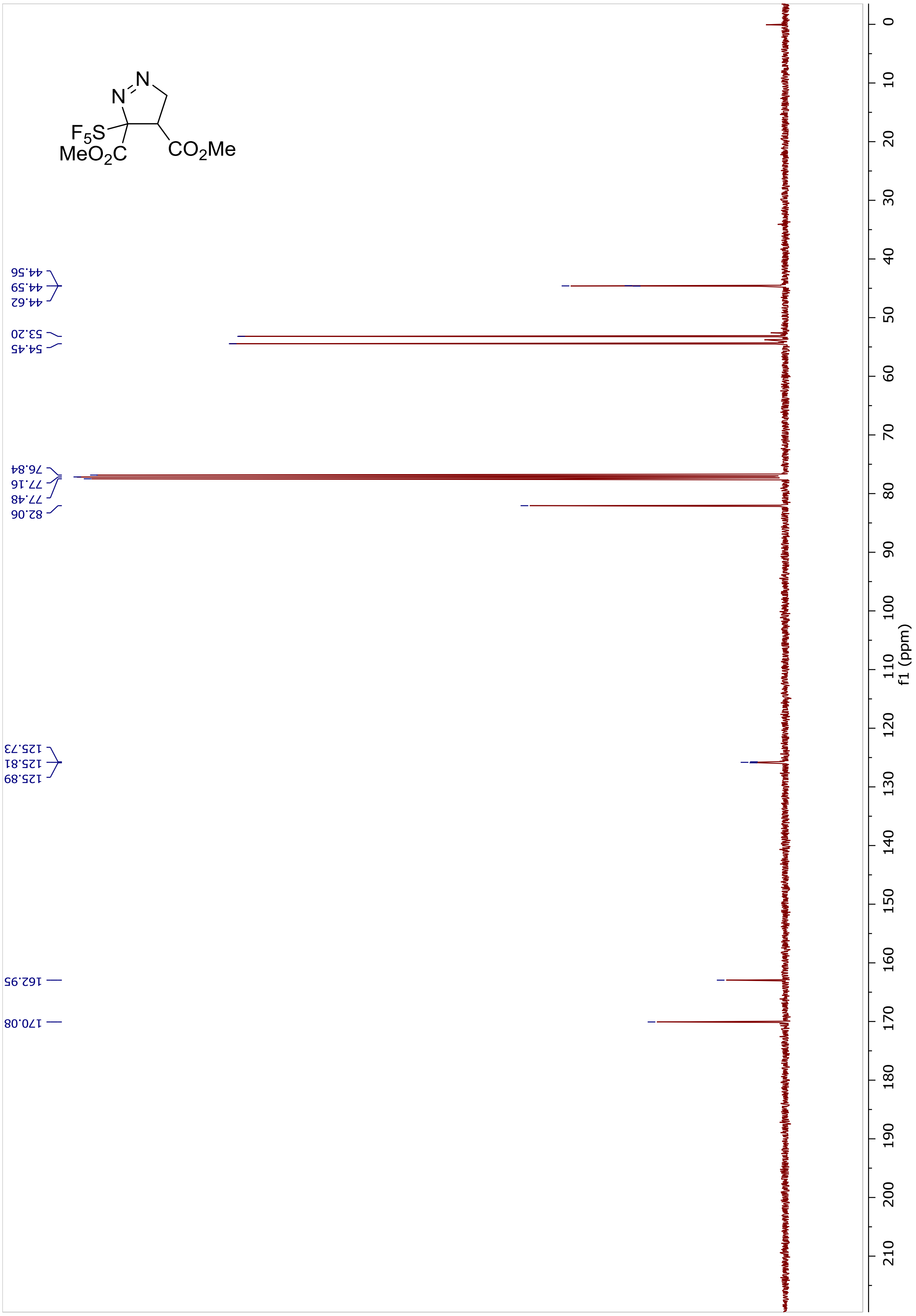

Dimethyl 3-(pentafluorosulfanyl)-4,5-dihydro-3*H*-pyrazole-3,4-dicarboxylate (22), ^{19}F NMR (376 MHz, $CDCl_3$)

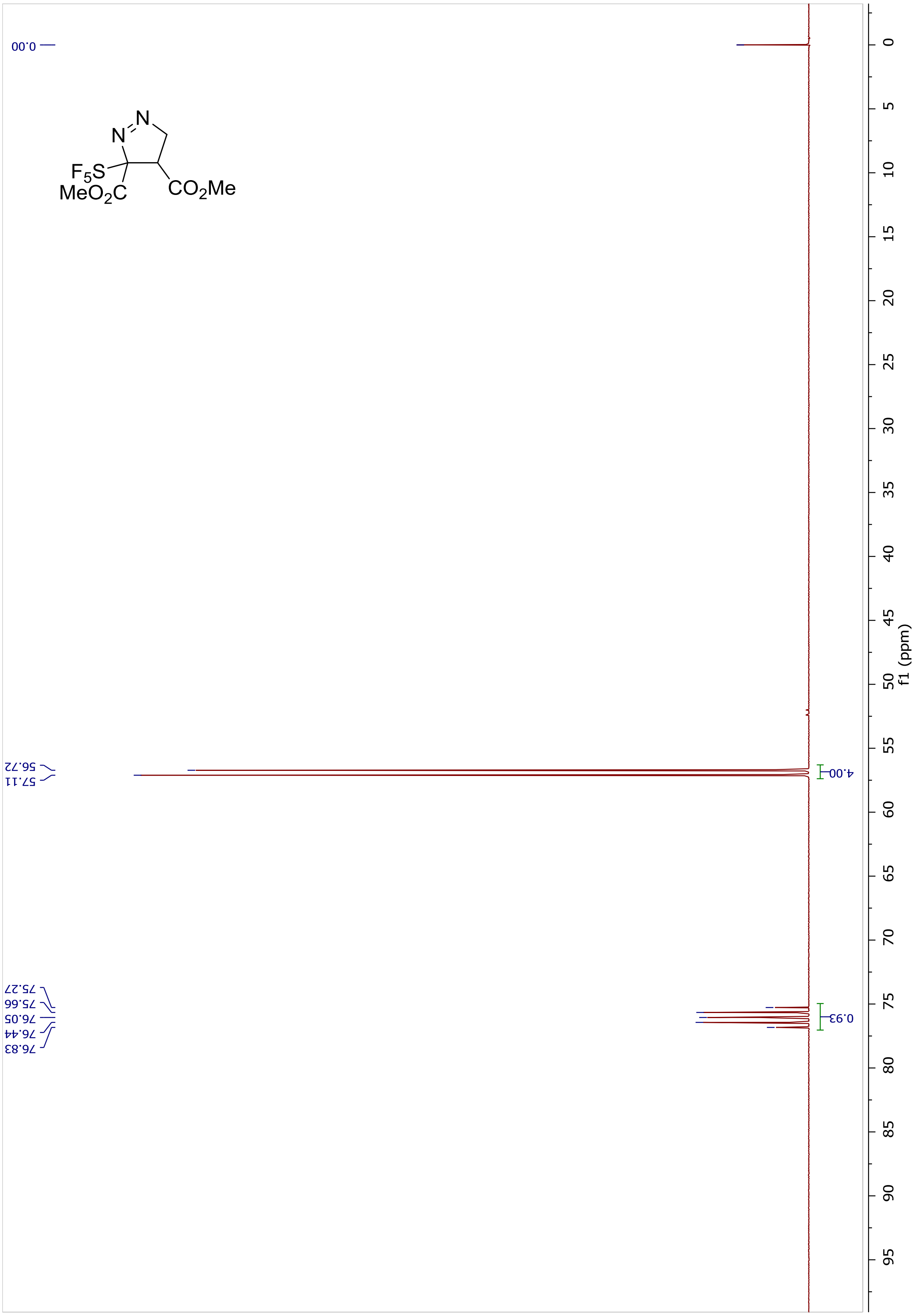

4. Computational data

All computations were performed in Gaussian 09, Revision D.01 [3]. The geometry optimizations were done at the density functional theory (DFT) level in the gas phase employing restricted B3LYP functional [4] and aug-cc-pVDZ basis set [5, 6] for all atoms. Frequency analyses were carried out at 298.15 K for all geometries, confirming either true energy minima (no imaginary vibrational frequency) or transition states (one imaginary vibrational frequency).

**Compound**	**cyclopentadiene**
Single-point energy (au)	–194.1239093
Gibbs energy (au)	–194.058781
Imaginary frequency (cm^{-1})	none

```
0 1
 C                  0.00026500    1.21887800    0.00042800
 H                  0.00040200    1.88461400   -0.88004800
 H                  0.00041600    1.88368500    0.88163400
 C                 -1.18122900    0.28415500   -0.00042600
 H                 -2.21893900    0.61195800   -0.00065300
 C                 -0.73567000   -0.99403900    0.00013600
 H                 -1.35517600   -1.89016300    0.00026300
 C                  1.18135300    0.28364200   -0.00045200
 H                  2.21920400    0.61099500   -0.00069600
 C                  0.73523800   -0.99435800    0.00017700
 H                  1.35435400   -1.89075100    0.00032700

 1 2 1.0 3 1.0 4 1.0 8 1.0
 2
 3
 4 5 1.0 6 2.0
 5
 6 7 1.0 10 1.0
 7
 8 9 1.0 10 2.0
 9
 10 11 1.0
 11
```

**Compound**	**12**
Single-point energy (au)	–1278.262948
Gibbs energy (au)	–1278.210168
Imaginary frequency (cm^{-1})	none

```
0 1
 C                 -2.68358800   -0.92678000    0.00000900
 C                 -1.19128700   -1.03213500    0.00000600
 C                 -0.41073100    0.05708500    0.00001500
```

C	-0.99233200	1.44709100	0.00003800
C	-2.48267600	1.51496900	0.00000000
C	-3.27316600	0.42883500	-0.00000600
H	-0.79404500	-2.04511600	-0.00000500
H	-2.87912400	2.53108000	-0.00001500
H	-4.36164300	0.48983600	-0.00002500
O	-0.33731800	2.47081300	-0.00001400
O	-3.36575600	-1.93994700	-0.00000700
S	1.43282700	-0.18503000	0.00000000
F	1.63751700	0.94073700	-1.17189700
F	1.63752600	0.94065000	1.17197900
F	3.05393800	-0.40906600	-0.00001400
F	1.33200500	-1.35823800	1.16220400
F	1.33199800	-1.35815200	-1.16229000

1 2 1.0 6 1.0 11 2.0
2 3 2.0 7 1.0
3 4 1.0 12 1.0
4 5 1.0 10 2.0
5 6 2.0 8 1.0
6 9 1.0
7
8
9
10
11
12 13 1.0 14 1.0 15 1.0 16 1.0 17 1.0
13
14
15
16
17

**Compound**	**TS-*endo*-16a**
Single-point energy (au)	-1472.36652796
Gibbs energy (au)	-1472.224437
Imaginary frequency (cm^{-1})	-427.5435

0 1

C	-0.26990800	-0.69693700	-1.24359500
C	-1.48272100	1.86461600	-0.48079600
C	-0.08864600	1.49842900	-0.06769100
C	0.46294000	0.32492700	-0.40782500
H	0.45041700	2.26108600	0.49000100
O	-1.85143600	3.02862500	-0.37519000
O	0.26972200	-1.62858300	-1.81099800
C	-2.31959800	0.77462700	-0.99154800
H	-3.24058400	1.09379600	-1.47607200
C	-1.73355000	-0.46425800	-1.33631900
H	-2.19038200	-1.06758800	-2.11967800

C	-3.40295900	0.21126100	0.87971400
H	-4.08793100	1.04738600	1.00235200
C	-2.42155200	-1.77330600	0.22334000
H	-2.23993200	-2.75517800	-0.20911300
C	-1.73562500	-1.27596000	1.35377500
H	-0.82294300	-1.69706600	1.76885600
C	-2.32638800	-0.06831100	1.74337300
H	-1.94610200	0.60249000	2.51060100
C	-3.77218200	-1.09031800	0.20839900
H	-4.44438200	-1.64781200	0.88538300
H	-4.26394000	-1.01092100	-0.76520500
S	2.20134900	-0.01804800	0.16505100
F	2.70178000	-0.43821400	-1.33382600
F	1.82637800	-1.58069000	0.52143200
F	3.72011000	-0.32473500	0.70273300
F	2.68770300	1.53857000	-0.11744300
F	1.81503300	0.40880500	1.72450300

1 4 1.0 7 2.0 10 1.0
2 3 1.0 6 2.0 8 1.0
3 4 2.0 5 1.0
4 23 1.0
5
6
7
8 9 1.0 10 1.5
9
10 11 1.0
11
12 13 1.0 18 1.5 20 1.0
13
14 15 1.0 16 1.5 20 1.0
15
16 17 1.0 18 1.5
17
18 19 1.0
19
20 21 1.0 22 1.0
21
22
23 24 1.0 25 1.0 26 1.0 27 1.0 28 1.0
24
25
26
27
28

**Compound**	**TS-*exo*-16a**
Single-point energy (au)	-1472.36243700
Gibbs energy (au)	-1472.220704

Imaginary frequency (cm^{-1}) -429.2670

0 1

C	1.27731800	1.91294200	-0.47746400
C	0.22556400	-0.77145200	-1.03942800
C	-0.60163500	0.29318800	-0.35431500
C	-0.12275900	1.51920200	-0.10452100
H	-0.71874500	2.30149700	0.36032100
O	-0.23889200	-1.80915400	-1.47464600
O	1.59571300	3.09481500	-0.40304000
C	1.67072300	-0.44794200	-1.18171100
H	2.13733700	-1.06465000	-1.94542300
C	2.17864700	0.84684200	-0.92905700
H	3.05660700	1.20134200	-1.46151900
C	3.31627700	0.48363000	0.98873300
H	3.45433100	1.48673800	1.38621400
C	2.45859400	-1.60361100	0.48783000
H	1.84701400	-2.50335500	0.47320600
C	3.82122400	-1.56607600	0.11695300
H	4.34379900	-2.35119200	-0.42588100
C	4.33640100	-0.29882400	0.41290100
H	5.32401700	0.06398000	0.13525700
C	2.25399000	-0.47202500	1.46551600
H	2.55182500	-0.83699700	2.46495100
H	1.24074700	-0.07676800	1.55276000
S	-2.34666300	-0.08220100	0.17476300
F	-1.91340100	-1.56717400	0.73072300
F	-2.74158200	-0.69850900	-1.28790500
F	-2.06916400	0.54255100	1.68882200
F	-3.87604700	-0.40873800	0.66787600
F	-2.89213400	1.40232500	-0.31365400

1 4 1.0 7 2.0 10 1.0
2 3 1.0 6 2.0 8 1.0
3 4 2.0 23 1.0
4 5 1.0
5
6
7
8 9 1.0 10 1.5
9
10 11 1.0
11
12 13 1.0 18 1.5 20 1.0
13
14 15 1.0 16 1.5 20 1.0
15
16 17 1.0 18 1.5
17
18 19 1.0

19
20 21 1.0 22 1.0
21
22
23 24 1.0 25 1.0 26 1.0 27 1.0 28 1.0
24
25
26
27
28

**Compound**	**TS-*endo*-16b**
Single-point energy (au)	-1472.35844744
Gibbs energy (au)	-1472.215979
Imaginary frequency (cm^{-1})	-347.5485

0 1

C	-0.38110600	0.78588100	1.46641100
C	-1.93492200	1.33926500	-0.98391000
C	-2.32247200	1.90431300	0.32778300
C	-1.61961400	1.62447200	1.44032200
H	-3.18457300	2.57238900	0.33367900
H	-1.88858800	2.04131700	2.41234300
O	-2.47213700	1.68668300	-2.02350400
O	0.21081800	0.64587800	2.52901100
C	-0.86440300	0.27785900	-0.97019600
H	-0.40376500	0.17401500	-1.95033500
C	0.00413800	0.20648800	0.15880800
C	-1.99291400	-1.30262200	-1.04222900
H	-2.39823600	-1.23949600	-2.05173600
C	-0.90501700	-2.14303700	0.82061200
H	-0.12377700	-2.52011700	1.47457600
C	-2.14717700	-1.65007500	1.23016600
H	-2.47721900	-1.55232400	2.26150200
C	-2.84516600	-1.20389200	0.10203600
H	-3.82792500	-0.73802100	0.11125000
C	-0.94969100	-2.34309800	-0.66049300
H	-1.39753700	-3.33501700	-0.85469800
H	0.00312700	-2.29668800	-1.18057000
S	1.83495400	0.07217400	-0.18744100
F	1.62231600	-1.05801300	-1.39802200
F	1.76026600	1.26348500	-1.34622700
F	2.23488500	1.20545600	0.92234400
F	3.44486300	-0.04142000	-0.52827300
F	2.06876300	-1.13558200	0.91274900

1 4 1.0 8 2.0 11 1.0
2 3 1.0 7 2.0 9 1.0
3 4 2.0 5 1.0
4 6 1.0

5
6
7
8
9 10 1.0 11 1.5
10
11 23 1.0
12 13 1.0 18 1.5 20 1.0
13
14 15 1.0 16 1.5 20 1.0
15
16 17 1.0 18 1.5
17
18 19 1.0
19
20 21 1.0 22 1.0
21
22
23 24 1.0 25 1.0 26 1.0 27 1.0 28 1.0
24
25
26
27
28

**Compound**	**TS-*exo*-16b**
Single-point energy (au)	-1472.35804469
Gibbs energy (au)	-1472.215611
Imaginary frequency (cm^{-1})	-355.2821

0 1

C	-2.19374300	-0.96018200	-1.09547600
C	-0.43760000	-1.26214700	1.24816300
C	-1.68946500	-2.06022700	1.04751500
C	-2.48433900	-1.94689600	-0.03027300
H	-1.89874800	-2.76659400	1.85224300
H	-3.37157100	-2.56432000	-0.17381700
O	0.20734500	-1.43014000	2.27479800
O	-2.86974200	-0.90033900	-2.11111400
C	-0.10157100	-0.31904200	0.15450100
C	-1.03006900	-0.02236500	-0.87878800
H	-0.63621300	0.32209500	-1.82989900
C	-2.06045600	1.60890600	-0.34810800
H	-2.93548500	1.54926000	-0.99349800
C	-0.74054100	1.72495000	1.55235900
H	-0.28741500	1.48090700	2.50956500
C	-0.26579300	2.66208600	0.63592300
H	0.65330800	3.23203600	0.73635000
C	-1.08790500	2.63413200	-0.49895800
H	-0.92122600	3.20311600	-1.41127500

C	-2.13226500	1.34574100	1.14764400
H	-2.82957800	2.08457600	1.58084600
H	-2.47737700	0.35867900	1.45773100
S	1.70602300	-0.16660400	-0.28288300
F	1.47295800	-0.93197400	-1.74316500
F	2.09929400	-1.61351400	0.38155800
F	3.29212800	-0.02453900	-0.71505100
F	2.10788600	0.61021500	1.11424500
F	1.47769100	1.29092500	-1.05076400

1 4 1.0 8 2.0 10 1.0
2 3 1.0 7 2.0 9 1.0
3 4 2.0 5 1.0
4 6 1.0
5
6
7
8
9 10 1.5 23 1.0
10 11 1.0
11
12 13 1.0 18 1.5 20 1.0
13
14 15 1.0 16 1.5 20 1.0
15
16 17 1.0 18 1.5
17
18 19 1.0
19
20 21 1.0 22 1.0
21
22
23 24 1.0 25 1.0 26 1.0 27 1.0 28 1.0
24
25
26
27
28

**Compound**	***endo*-16a**
Single-point energy (au)	–1472.40873315
Gibbs energy (au)	–1472.262876
Imaginary frequency (cm^{-1})	none

0 1

C	-0.27838600	-0.95838500	-0.79430600
C	-1.40124100	1.79279000	-0.45019800
C	0.02220100	1.44895200	-0.15689400
C	0.52716700	0.21527200	-0.30697800
H	0.63221800	2.28612700	0.17634800

O	-1.74409400	2.96137400	-0.38631600
O	0.20295400	-2.04260700	-1.04645800
C	-2.36930400	0.69065600	-0.80226600
H	-2.86900300	1.00807500	-1.72721000
C	-1.77614900	-0.73694500	-0.96488200
H	-1.99823700	-1.12885600	-1.96585000
C	-3.48267800	0.48737600	0.31238700
H	-4.19936300	1.31159400	0.34806100
C	-2.61174300	-1.58676100	0.08466300
H	-2.53101100	-2.66270500	-0.08774000
C	-2.23757700	-1.08226100	1.47081100
H	-1.58372700	-1.60313000	2.16736900
C	-2.75682800	0.15027800	1.60702600
H	-2.61375300	0.83806100	2.43806500
C	-3.99348400	-0.91472800	-0.09370000
H	-4.73954500	-1.30527800	0.60820200
H	-4.38239400	-0.96576100	-1.12015900
S	2.33526200	-0.01388300	0.11478500
F	2.63218500	-0.81889500	-1.27928600
F	2.03002800	-1.41502600	0.91046400
F	3.91656000	-0.20198400	0.50143700
F	2.75497200	1.40444300	-0.63270000
F	2.15490700	0.81550500	1.53948200

1 4 1.0 7 2.0 10 1.0
2 3 1.0 6 2.0 8 1.0
3 4 2.0 5 1.0
4 23 1.0
5
6
7
8 9 1.0 10 1.0 12 1.0
9
10 11 1.0 14 1.0
11
12 13 1.0 18 1.0 20 1.0
13
14 15 1.0 16 1.0 20 1.0
15
16 17 1.0 18 2.0
17
18 19 1.0
19
20 21 1.0 22 1.0
21
22
23 24 1.0 25 1.0 26 1.0 27 1.0 28 1.0
24
25
26

27
28

**Compound**	***exo*-16a**
Single-point energy (au)	–1472.40809036
Gibbs energy (au)	–1472.261284
Imaginary frequency (cm^{-1})	none

0 1

C	1.26555300	1.84930000	-0.40502600
C	0.24855400	-0.92766500	-0.71570200
C	-0.61347400	0.22077000	-0.26449600
C	-0.15378600	1.47315100	-0.13104500
H	-0.79753600	2.29995600	0.16202100
O	-0.19558200	-2.01680700	-1.00969400
O	1.56118700	3.03236900	-0.40861500
C	1.75437700	-0.68252700	-0.78777800
H	2.05284800	-1.12701500	-1.74351400
C	2.29539100	0.76995600	-0.64524600
H	2.85809800	1.08193200	-1.53255900
C	3.28460800	0.65658500	0.59684400
H	3.54859300	1.62953900	1.01962400
C	2.48879900	-1.44726600	0.39962400
H	2.02423400	-2.40653400	0.64133000
C	3.95743700	-1.47474900	0.00537400
H	4.47494700	-2.34455700	-0.39466300
C	4.43091100	-0.22261700	0.12237200
H	5.41582200	0.14279900	-0.16201200
C	2.51249700	-0.34656600	1.48553100
H	3.08555800	-0.64691700	2.36966200
H	1.51829200	-0.00089800	1.79839000
S	-2.42053700	-0.06734800	0.11966400
F	-2.08132500	-1.45539700	0.92513500
F	-2.66374300	-0.88504600	-1.27631400
F	-2.29218400	0.77156600	1.54507700
F	-4.00040700	-0.30800300	0.47947500
F	-2.87370700	1.33405200	-0.63883400

1 4 1.0 7 2.0 10 1.0
2 3 1.0 6 2.0 8 1.0
3 4 2.0 23 1.0
4 5 1.0
5
6
7
8 9 1.0 10 1.0 14 1.0
9
10 11 1.0 12 1.0
11
12 13 1.0 18 1.0 20 1.0

13
14 15 1.0 16 1.0 20 1.0
15
16 17 1.0 18 2.0
17
18 19 1.0
19
20 21 1.0 22 1.0
21
22
23 24 1.0 25 1.0 26 1.0 27 1.0 28 1.0
24
25
26
27
28

**Compound**	***endo*-16b**
Single-point energy (au)	–1472.41015715
Gibbs energy (au)	–1472.263317
Imaginary frequency (cm^{-1})	none

0 1

C	0.37464700	-0.68193600	1.51867600
C	1.87943500	-1.33032700	-0.92925400
C	1.66310400	-2.30961000	0.16133600
C	0.96748900	-2.01516000	1.27575200
H	2.15694800	-3.27379700	0.03136400
H	0.88282900	-2.71951600	2.10434500
O	2.67079300	-1.57149000	-1.82732100
O	0.10407100	-0.30896500	2.64323700
C	1.10886900	-0.01973400	-0.92300800
H	0.52411900	-0.02917500	-1.84743600
C	0.18385800	0.25584800	0.30157800
C	2.01819300	1.26686500	-0.95828600
H	2.60108100	1.33407400	-1.87960000
C	0.52696900	1.74657800	0.67355600
H	-0.23044500	2.25053900	1.27090900
C	1.91014000	1.60361100	1.32658600
H	2.07834400	1.63367600	2.39920300
C	2.79121700	1.31692700	0.35508800
H	3.84933900	1.09118300	0.46886400
C	0.95004700	2.35212400	-0.68726300
H	1.39615500	3.34370900	-0.55444800
H	0.15646500	2.39807400	-1.43640200
S	-1.72204400	-0.02118200	-0.23738000
F	-1.57121600	0.99349500	-1.54924500
F	-1.32086700	-1.32754700	-1.19657100
F	-2.04620600	-1.07312800	1.00256700
F	-3.27423200	-0.24769700	-0.71813100

F	-2.26579400	1.23739700	0.68642300

1 4 1.0 8 2.0 11 1.0
2 3 1.0 7 2.0 9 1.0
3 4 2.0 5 1.0
4 6 1.0
5
6
7
8
9 10 1.0 11 1.0 12 1.0
10
11 14 1.0
12 13 1.0 18 1.0 20 1.0
13
14 15 1.0 16 1.0 20 1.0
15
16 17 1.0 18 2.0
17
18 19 1.0
19
20 21 1.0 22 1.0
21
22
23 24 1.0 25 1.0 26 1.0 27 1.0 28 1.0
24
25
26
27
28

**Compound**	***exo*-16b**
Single-point energy (au)	–1472.40900313
Gibbs energy (au)	–1472.261245
Imaginary frequency (cm^{-1})	none

0 1

C	-2.12020300	-1.02075000	-0.97733700
C	-0.39097500	-1.12054300	1.37592100
C	-1.04839400	-2.32877400	0.83168700
C	-1.85738800	-2.27934300	-0.24207500
H	-0.92456700	-3.23674200	1.42290100
H	-2.41379300	-3.15238700	-0.58622400
O	-0.08444500	-1.05345300	2.54916200
O	-3.01891900	-0.96677800	-1.80176000
C	-0.19750900	0.09849500	0.42802100
C	-1.26065500	0.20986600	-0.71009800
H	-0.79245000	0.43072500	-1.67240300
C	-2.10560100	1.47169200	-0.24785700
H	-3.10687100	1.47995400	-0.68477400

C	-0.41053600	1.41698900	1.25570700
H	0.11101600	1.39599000	2.21205000
C	-0.21368400	2.64069900	0.37218200
H	0.65610600	3.29026000	0.41616700
C	-1.22472800	2.68246800	-0.50846700
H	-1.35085800	3.37591800	-1.33723800
C	-1.96981300	1.36656200	1.28718800
H	-2.38099900	2.23422700	1.81411600
H	-2.38961700	0.44866500	1.71921800
S	1.60809500	-0.10921600	-0.31906800
F	1.06124800	-1.16589600	-1.48443500
F	1.97514500	-1.40769000	0.64525800
F	3.10663700	-0.28582800	-0.96470900
F	2.27994400	0.90680900	0.79832200
F	1.40884300	1.15466200	-1.36856600

1 4 1.0 8 2.0 10 1.0
2 3 1.0 7 2.0 9 1.0
3 4 2.0 5 1.0
4 6 1.0
5
6
7
8
9 10 1.0 14 1.0
10 11 1.0 12 1.0
11
12 13 1.0 18 1.0 20 1.0
13
14 15 1.0 16 1.0 20 1.0
15
16 17 1.0 18 2.0
17
18 19 1.0
19
20 21 1.0 22 1.0
21
22
23 24 1.0 25 1.0 26 1.0 27 1.0 28 1.0
24
25
26
27
28

5. References

1. Svendsen, A.; Boll, P. M. *J. Org. Chem.* **1975,** *40,* 1927–1932. doi:10.1021/jo00901a013
2. Brel, V. K. *Synthesis* **2006,** 339–343. doi:10.1055/s-2005-918508
3. Gaussian 09, Revision D.01, Frisch, M. J.; Trucks, G. W.; Schlegel, H. B.; Scuseria, G. E.; Robb, M. A.; Cheeseman, J. R.; Scalmani, G.; Barone, V.; Mennucci, B.; Petersson, G. A.; Nakatsuji, H.; Caricato, M.; Li, X.; Hratchian, H. P.; Izmaylov, A. F.; Bloino, J.; Zheng, G.; Sonnenberg, J. L.; Hada, M.; Ehara, M.; Toyota, K.; Fukuda, R.; Hasegawa, J.; Ishida, M.; Nakajima, T.; Honda, Y.; Kitao, O.; Nakai, H.; Vreven, T.; Montgomery, J. A., Jr.; Peralta, J. E.; Ogliaro, F.; Bearpark, M.; Heyd, J. J.; Brothers, E.; Kudin, K. N.; Staroverov, V. N.; Kobayashi, R.; Normand, J.; Raghavachari, K.; Rendell, A.; Burant, J. C.; Iyengar, S. S.; Tomasi, J.; Cossi, M.; Rega, N.; Millam, J. M.; Klene, M.; Knox, J. E.; Cross, J. B.; Bakken, V.; Adamo, C.; Jaramillo, J.; Gomperts, R.; Stratmann, R. E.; Yazyev, O.; Austin, A. J.; Cammi, R.; Pomelli, C.; Ochterski, J. W.; Martin, R. L.; Morokuma, K.; Zakrzewski, V. G.; Voth, G. A.; Salvador, P.; Dannenberg, J. J.; Dapprich, S.; Daniels, A. D.; Farkas, Ö.; Foresman, J. B.; Ortiz, J. V.; Cioslowski, J.; Fox, D. J. Gaussian, Inc., Wallingford CT, 2013.
4. Becke, A. D. *J. Chem. Phys.* **1993,** *98,* 5648–5652. doi:10.1063/1.464913
5. Dunning Jr., T. H. *J. Chem. Phys.* **1989,** *90,* 1007–1023. doi:10.1063/1.456153
6. Kendall, R. A.; Dunning Jr., T. H.; Harrison, R. J. *J. Chem. Phys.* **1992,** *96,* 6796–6806. doi:10.1063/1.462569
